# The effectiveness of the Mediterranean Diet for primary and secondary prevention of cardiovascular disease: An umbrella review

**DOI:** 10.1111/1747-0080.12891

**Published:** 2024-08-14

**Authors:** Laima W. Hareer, Yan Ying Lau, Frances Mole, Dianne P. Reidlinger, Hayley M. O'Neill, Hannah L. Mayr, Hannah Greenwood, Loai Albarqouni

**Affiliations:** ^1^ Faculty of Health Sciences and Medicine Bond University Gold Coast Queensland Australia; ^2^ Department of Nutrition and Dietetics Princess Alexandra Hospital Brisbane Queensland Australia; ^3^ Centre for Functioning and Health Research Metro South Health Brisbane Queensland Australia; ^4^ Faculty of Health Sciences and Medicine, Institute for Evidence‐Based Healthcare Bond University Gold Coast Queensland Australia

**Keywords:** cardiovascular disease, chronic disease, dietary, Mediterranean Diet, nutrition, umbrella review

## Abstract

**Aims:**

This study aimed to review meta‐analyses of randomised controlled trials that evaluated the effectiveness of the Mediterranean Diet for the primary and secondary prevention of cardiovascular disease.

**Methods:**

Five databases (Medline, Embase, Cochrane, CINAHL and ProQuest) were searched from inception to November 2022. Inclusion criteria were: (i) systematic review of randomised controlled studies with metanalysis; (ii) adults ≥18 years from the general population with (secondary prevention) and without (primary prevention) established cardiovascular disease; (iii) Mediterranean Diet compared with another dietary intervention or usual care. Review selection and quality assessment using AMSTAR‐2 were completed in duplicate. GRADE was extracted from each review, and results were synthesised narratively.

**Results:**

Eighteen meta‐analyses of 238 randomised controlled trials were included, with an 8% overlap of primary studies. Compared to usual care, the Mediterranean Diet was associated with reduced cardiovascular disease mortality (*n* = 4 reviews, GRADE low certainty; risk ratio range: 0.35 [95% confidence interval: 0.15–0.82] to 0.90 [95% confidence interval: 0.72–1.11]). Non‐fatal myocardial infarctions were reduced (*n* = 4 reviews, risk ratio range: 0.47 [95% confidence interval: 0.28–0.79] to 0.60 [95% confidence interval: 0.44–0.82]) when compared with another active intervention. The methodological quality of most reviews (*n* = 16/18; 84%) was low or critically low and strength of evidence was generally weak.

**Conclusions:**

This review showed that the Mediterranean Diet can reduce fatal cardiovascular disease outcome risk by 10%–67% and non‐fatal cardiovascular disease outcome risk by 21%–70%. This preventive effect was more significant in studies that included populations with established cardiovascular disease. Better quality reviews are needed.

## 1 INTRODUCTION

Cardiovascular disease (CVD) is the leading cause of death globally.^1^ From 1990 to 2019, CVD cases rose from 270 million to 523 million. Additionally, CVD mortality increased from 12.1 million in 1990 to 18.6 million in 2019, accounting for 32% of all deaths worldwide. Of these deaths, approximately 85% were due to heart attacks and strokes, highlighting the importance of preventing CVD.^2^ Most CVD conditions can be prevented by modifying behavioural risk factors such as an unhealthy diet and optimising healthy eating interventions.^1^

The effectiveness of the Mediterranean Diet (MedDiet) in primary and secondary prevention of CVD has acquired considerable attention, particularly since the seven countries' study conducted in the 1950s.^3^ This study compared dietary habits across seven countries and revealed that populations in the Mediterranean region, encompassing countries such as Greece and Italy, exhibited lower CVD mortality rates compared to the United States of America (US) and Northern European countries.^3^, ^4^ The MedDiet has since been a focal point in cardiovascular health research and considered one of the healthiest dietary patterns in the world endorsed in the 2020–2025 American Dietary Guidelines.^4^, ^5^, ^6^ Although regional dietary habits have evolved since the 1950s; the main characteristics of the Mediterranean‐style diet remain. These include extra‐virgin olive oil as the primary dietary fat source, high consumption of vegetables, fruits, nuts, legumes and unrefined cereals, moderate consumption of fish and shellfish, moderate consumption of red wine within meals only, moderate consumption of fermented dairy products, such as cheese and yoghurt, low consumption of unfermented full‐fat dairy products, low consumption of meat and very low consumption of red meat and processed meat.^4^, ^5^

A large body of literature exists regarding adherence to MedDiet and CVD outcomes, including systematic reviews of observational studies.^7^ The MedDiet is inversely associated with all‐cause mortality, CVD mortality, coronary heart disease (CHD) and stroke incident risk.^8^, ^9^, ^10^, ^11^, ^12^ The MedDiet's potential beneficial effects on reducing CVD risk are likely due to the combination of dietary components, particularly the high content of polyunsaturated fatty acids, fibre and polyphenols, which collectively modify CVD risk factors, including blood lipid and glucose levels, blood pressure and body weight.^8^, ^9^, ^13^, ^14^

There is also a significant amount of randomised controlled trial (RCT) data on the effects of the MedDiet and CVD end points or risk factors. A small number of these represent large landmark trials, which are split across primary and secondary CVD prevention outcomes. A well‐cited MedDiet RCT targeting people at high risk of CVD (primary prevention) is the PREDIMED study, which recruited almost 4500 participants and compared two MedDiet intervention arms with a low‐fat control diet, finding a reduction of around 30% in cardiovascular events for the MedDiet arms compared to the control group.^15^ With regards to secondary prevention, the recent CORDIOPREV study was conducted in people with established CVD, and demonstrated a 27% reduction in risk of major cardiovascular events with MedDiet in comparison to a low‐fat diet.^16^ The Lyon Diet Heart Study also provided causative evidence for the beneficial effects of MedDiet in people with established CVD, showing a 50%–70% reduction in recurrent CVD events.^17^

Both PREDIMED^15^ and the Lyon Diet Heart Study^17^ have been criticised for methodological limitations, some avoidable and others reflecting the inherent difficulties in conducting a gold standard RCT with a complex intervention such as dietary change. Of particular interest are RCTs testing the effectiveness of the MedDiet in non‐Mediterranean populations, given that Spain was the setting for PREDIMED and CORDIOPREV, and Southeastern France was the setting for the Lyon Diet Heart Study.^15^, ^16^, ^17^ Baseline diets in Mediterranean populations are likely to already reflect several attributes of the MedDiet,^18^ and there is likely to be higher acceptability of MedDiet food components in Mediterranean than in non‐Mediterranean settings.

A previous umbrella review of 13 meta‐analyses of observational studies and 16 meta‐analyses of RCTs involving 12 800 000 participants examined the health benefits of the MedDiet on 37 different health outcomes, including CVD.^19^ The findings emphasised the importance of adhering to the MedDiet for both primary and secondary prevention of CVD. However, it has been over 7 years since the search for this umbrella review was conducted, and since then, numerous more systematic reviews have been published on the impact of MedDiet on CVD outcomes and risk factors. Further, the majority of systematic reviews have not focused solely on gold standard RCT evidence, with observational study designs dominating and summaries of effects of RCTs showing significant overlap between reviews.^19^

This umbrella review aims to summarise the characteristics of systematic reviews of RCTs and their reported effects of MedDiet compared to any other diet, no intervention or usual diet on the primary and secondary prevention of CVD, including all‐cause mortality, cardiovascular events and clinical and biochemical risk factors for CVD. A further aim was to appraise the systematic review quality and to describe the quality and certainty of the evidence reported.

## 2 METHODS

An umbrella review of systematic reviews of RCTs evaluating the effect of MedDiet on the primary and secondary prevention of CVD was conducted. An umbrella review was considered appropriate due to the large number of published systematic reviews and the need to assess the strength of available evidence reported in these reviews. RCTs were selected as the study design, as they are often considered the gold standard in evidence‐based research for guiding clinical recommendations^20^ due to their rigorous design and ability to establish causation more reliably than observational studies.^21^ It is recognised that observational studies are often considered in developing guidance from nutrition research; however, few would argue that controlled intervention studies provide greater certainty of findings.^22^ Given the large number of MedDiet systematic reviews published over the last 5 years, sufficient evidence was expected in a new umbrella review without incorporating observational studies, as noted in more recently published systematic reviews.^19^

This review is reported according to the Preferred Reporting Items for Systematic Reviews and Meta‐Analyses (PRISMA) statement^23^ and reporting guidelines for umbrella reviews.^24^ The protocol was registered on PROSPERO (CRD42022376396) and Open Science Framework (OSF) (https://doi.org/10.17605/OSF.IO/7XPEB).

Five electronic databases, The Cochrane Database of Systematic Reviews, Medline (PubMed), Elsevier (Embase) and Cumulative Index of Nursing and Allied Health Literature (CINAHL), were searched from inception to 4 November 2022. The ProQuest database (Dissertations and Thesis tab) was also searched to allow the inclusion of grey literature (theses) reporting unpublished systematic reviews that met the criteria. The search strategy was designed in PubMed with information specialists and used a combination of free text and MeSH terms and keywords. There was no limitation on language or publication date. The search strategy was translated to search the other databases using Polyglot^25^ (Supporting Information S1: Table 1).

Pairs of reviewers screened titles, abstracts and full texts of potentially eligible search results independently using Covidence.^26^ Discrepancies were resolved by consensus or discussion with another reviewer.

Studies were included if they met the following criteria: (1) systematic reviews (as defined by Page et al.)^27^ of RCTs that included at least one study reporting on the MedDiet; (2) adults ≥18 years aged from the general population with (secondary prevention) and without (primary prevention) established CVD; (3) comparator groups with another dietary intervention other than the MedDiet, such as Dietary Approaches to Stop Hypertension (DASH), a diet with a low glycaemic index, no intervention, or usual care; (4) reported any outcomes of interest including all‐cause mortality or CVD outcomes (cardiovascular mortality, major cardiovascular events, or surrogate markers of CVD risk, e.g. weight, BMI, blood pressure, blood lipids and blood glucose); (5) outcomes were meta‐analysed. CVD was defined by a spectrum of conditions that impact the heart and blood vessels. No specific inclusion criteria (e.g. food‐based principles or macronutrient targets) were applied concerning the MedDiet intervention outside of the systematic review needing to have included one or more studies of dietary interventions referred to as ‘Mediterranean’. It was not practical within the umbrella review methodology to apply a specific definition for the MedDiet to the criteria as many of the systematic reviews did not report on this in their inclusion criteria or reporting of study characteristics.

Umbrella reviews returned in the search were excluded. Reviews that examined health outcomes unrelated to CVD, such as dementia, Alzheimer's, cancer, pregnancy, prenatal, and human immunodeficiency virus, were excluded during the screening process. Systematic reviews that only included one eligible primary study (Supporting Information S1: Table 3) and overlapped in reporting (i.e. the study was reported in another review of higher quality) or could not be translated into English were also excluded.

After piloting extraction for five included systematic reviews, three reviewers refined and used a prospectively developed standardised form to extract the included studies' characteristics and outcomes. Data were extracted by one reviewer and reviewed for accuracy by a second reviewer, with disagreements resolved through consensus or by another reviewer. The following study characteristics and outcomes data were extracted from each included review: characteristics of included systematic reviews (study aims, N studies, N analysed, types of studies (RCTs), country, year, N participants; Mediterranean Diet intervention and control diet characteristics (if reported); study duration; setting of study (i.e. community/outpatient hospital), and search methods), outcomes of data synthesis, that is, metanalysis (N intervention, N control, *p* value, pooled effect estimate and heterogeneity [*I* ^2^%]). Conflicts of interest of investigators and quality assessment methods were also extracted. Where a review included RCTs and other study designs, data from RCTs only were extracted if possible.

The effect estimates and 95% CI (along with the number of studies and *I* ^2^ statistics for heterogeneity) for each primary and secondary outcome were extracted from the meta‐analyses of included systematic reviews. Risk ratios (RRs) or odds ratios (ORs) with 95% CIs were reported for categorical outcomes. Mean differences (MDs) with 95% CIs were extracted for continuous outcomes reported on the same scale. Standardised mean difference (SMD) with 95% CI for continuous outcomes was also extracted for studies using different scales. All original studies reported in more than one systematic review were highlighted. The 95% CIs were calculated using fixed and random effects models.^23^ Outcome data from each included review was summarised in tables according to the target population (primary and/or secondary prevention) and described narratively. Primary outcomes were grouped as fatal or non‐fatal; surrogate outcomes were grouped into vascular, blood lipid, blood glucose and anthropometric measurements in the summary table.

The web‐based tool (metaumbrella.org) was used and the guidelines for analysing umbrella reviews were followed. Included meta‐analyses were analysed if two or more of the included systematic reviews reported data for the same outcome and population. We extracted data from individual RCTs reported in each included meta‐analysis. Extracted data from individual RCTs included sample size, number of events, effect size and uncertainty around the effect estimates (CIs or standard errors).

Quality assessments of the reviews were conducted independently by two reviewers using the Assessment of Multiple Systematic Review (AMSTAR‐2) tool.^24^ The AMSTAR‐2, a ‘yes or no’ checklist, comprised 16 questions designed to assess the risk of bias with gradings of ‘high’, ‘moderate’, ‘low’ or ‘critically low’. Disagreements regarding all quality assessments were determined by consensus through discussion or by involving another reviewer.

The Grading of Recommendations, Assessment, Development and Evaluations (GRADE) was extracted and recorded from the included systematic reviews (where it was assessed) following GRADE or Cochrane guidelines.^23^, ^28^

Overlap of primary studies in the included meta‐analyses was calculated and reported as a percentage. An equation was used to calculate the overlap percentage: $\frac{N-r}{\left(r*c\right)-r}*100\%$ (where *N* represents the total number of primary studies including double counting of overlapping studies, *r* is the number of primary studies not including double counting of overlapping studies and *c* is the total number of systematic reviews).^29^

## 3 RESULTS

**FIGURE 1:**
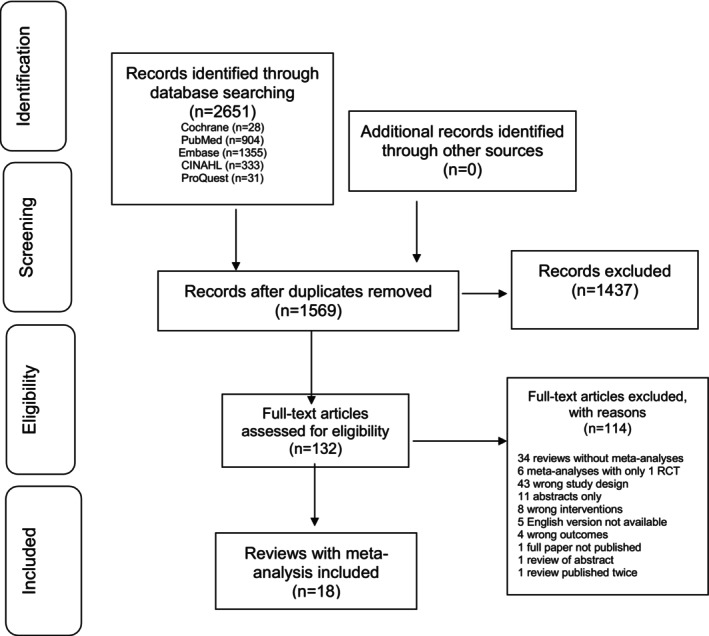
PRISMA flow chart for search strategy exploring the effects of the MedDiet on cardiovascular disease end points and surrogate outcomes. MedDiet, Mediterranean Diet; PRISMA, Preferred Reporting Items for Systematic Reviews and Meta‐Analyses.

Initial database and other searches yielded 2651 articles. After eliminating duplicates, 1437 articles were excluded based on their title and abstract, and 114 based on full‐text assessment. The remaining 18 articles met eligibility for data extraction and analysis (Figure 1). Reasons for study exclusion at the full‐text review stage were recorded (Supporting Information S1: Table 2).

The 18 meta‐analyses included a total of 238 RCTs (range: 3–72 RCTs) and total *n* of 197 965 participants. Although 133 primary studies were included in ≥2 systematic reviews, a moderate overlap^29^, ^30^ was calculated at 8% (Supporting Information S1: Table 2).

Thirteen meta‐analyses^13^, ^31^, ^32^, ^33^, ^34^, ^35^, ^36^, ^37^, ^38^, ^39^, ^40^, ^41^, ^42^ evaluated the effect of the MedDiet on the primary prevention of CVD (i.e. participants without established CVD), whereas the remaining meta‐analyses^11^, ^43^, ^44^, ^45^ reported the effect of MedDiet on primary and secondary prevention of CVD (i.e. participants with and without established CVD) (Table 1). One recently updated Cochrane SLR with 30 included‐RCTs reported their results separately for primary and secondary CVD prevention.^46^ The MedDiet (see Table 1 for descriptions of MedDiet interventions where reported in the reviews) was compared to various comparator diets, including low fat, Atkins, DASH, low carbohydrate, American Diabetes Association, prudent and usual, among others. The duration of included RCTs within the meta‐analyses ranged from 3 months to 4.8 years. Of the 18 meta‐analyses, 15 reported on the countries or regions where RCTs had been conducted, which included countries in the Mediterranean and non‐Mediterranean regions.^11^, ^13^, ^34^, ^35^, ^36^, ^37^, ^38^, ^39^, ^40^, ^42^, ^43^, ^44^, ^45^, ^46^ These studies covered specific Mediterranean nations—Spain, Italy and Greece—as well as a variety of non‐Mediterranean countries, including India, the UK, the US, Canada, Australia, New Zealand, Hong Kong, Brazil, Iran, China, and other European countries. The remaining three reviews did not provide information regarding the locations where the studies were conducted.^31^, ^32^, ^33^

**TABLE 1:** Characteristics of included meta‐analyses of randomised controlled trials of Mediterranean Diet (MedDiet) on cardiovascular disease outcomes.

First author, Year (Reference)	Included RCTs (*n*)	Included population (*n*)	Intervention^a^ (*n*)^b^	Outcomes reported	Follow up duration (median)	SR quality (AMSTAR‐2)
*Country/ies*			Comparator (*n*)			
S**tudies of MedDiet in primary CVD prevention**						
Ajala, 2013^31^ *NR*	4	Adults with T2DM (1926)	(I) MedDiet (Traditional and LC) (NR) (C) Any other diet (usual care, ADA) (NR)	HbA1c, weight loss, HDL, LDL, TG	NR	Critically low
Becerra‐Tomas, 2020^13^ *France, Spain, India*	3	Adults with type 1 diabetes or T2DM (9052)	(I) MedDiet (5705) (C) Compared to LF and LC diet (3347)	CVD incidence, CHD, MI, and stroke, mortality	27mo	Low
Carter, 2014^32^ *NR*	11	Free‐living adults at high risk or have diabetes (1790)	(I) All major components of the MedDiet (i.e. a high intake of fruit, vegetables, whole grains and monounsaturated fatty acids, regular fish and nut intake, moderate poultry and alcohol consumption and a low intake of red meat and saturated fatty acids) (1028) (C) Any other diet (ADA, LF, low GI, LC, MedDiet, usual care, Palaeolithic) (757)	FBG, fasting insulin, HbA1c	NR	Critically low
Cowell, 2021^33^ *NR*	19	Adults aged ≥18y (4137)	(I) MedDiet (defined as such by the authors of each study) alone or in combination with other lifestyle, clinical or pharmacological intervention (e.g. exercise) (NR) (C) LF diet, Habitual diet, Prudent diet, Palaeolithic diet, Central European, vegan diet, low GI diet, and energy restricted LF diet (NR)	SBP, DBP	NR	Critically low
Fatima, 2022^34^ *Italy, Spain, Sweden, Australia, US, UK, Brazil*	15	Adults aged ≥18y who are healthy or with cardiovascular risk factors (2735)	(I) MedDiet (which was defined as a MedDiet by the authors of each study) administered alone or with any other intervention if a comparable and valid control group was present (NR) (C) Any control diet (NR)	Endothelial function	NR	Critically low
Gay, 2016^35^ *Italy, Spain, US*	4	Adults aged ≥19y (5121)	(I) Dietary pattern change(s) (NR) (C) Control diet, advice only, or standard follow up (NR)	SBP, DBP	24mo	Critically low
Gibbs, 2021^36^ *Australia, Spain, Greece, Italy*	8	Adults aged ≥18y (5346)	(I) Plant‐based diet (dietary pattern that supports high consumption of fruits, vegetables, whole grains, legumes, nuts, and seeds, may often limit consumption of most or all animal products) (NR) (C) Any standardised control diet (NR)	SBP, DBP	NR	Low
Huo, 2015^37^ *US, Spain, Greece, Israel, Italy, Australia*	9	Adult patients T2DM (1144)	(I) MedDiet (NR) (C) Usual care, LF diet, high carbohydrate, ADA diet, usual diet (NR)	HbA1c, FBG, fasting insulin, HOMA‐IR, Anthropometric measurements, Blood lipids, BP	NR	Critically low
Ndanuko, 2016^38^ *US, Italy, France, Spain*	5	Adults aged >19y (1359)	(I) Dietary patterns (NR) (C) Usual care, Prudent diet, LF diet (NR)	BP	NR	Critically low
Pan, 2019^39^ *US, Sweden, Italy, Spain, Greece*	6	Patients with T2DM (589)	(I) MedDiet (high intake of fruits, vegetables, whole grains, nuts, beans and seeds; foods fresh and seasonal, minimal added sugars, olive oil as the main fat, moderate cheese and yoghurt, and limited red meat) (304) (C) Between intervention diet comparison (LF, and LC) (285)	Glycaemic control, CVD risk factors, and weight loss	NR	Critically low
Papadaki, 2020^40^ *Italy, UK, Spain, Canada, France, Greece, Australia, India, Germany, Norway, Netherlands*	72	Adults aged >18 years who were non‐pregnant, non‐lactating, and free of conditions that might affect their ability to eat certain foods (56 666)	(I) Whole MedDiet or Mediterranean‐style diet, with or without physical activity (physical activity was equally promoted in the control group) (35 480) (C) No treatment, usual care, or advice to follow a different diet (23 581)	Metsyn incidence, components & risk factors; incidence and/or mortality from related comorbidities	NR	Low
Rees, 2013^41^ *US, Italy, Finland, Spain, Norway, Iran, UK*	11	Adults of all ages (18 years or more) from the general population and those at high risk of CVD (52 026)	(I) Mediterranean‐style dietary pattern or provision of dietary factors relevant to the MedDiet (high intake of monosaturated fats (olive oil), legumes, grains/cereals, vegetables, moderate consumption of milk and dairy products, low to moderate intake of red wine, and low consumption of meat and meat products, increased consumption of fish) (20 043) (C) No intervention or minimal intervention (e.g. leaflet to follow a dietary pattern with no person‐to‐person intervention or reinforcement) (29 667)	CVD mortality, All‐cause mortality, non‐fatal end points, blood lipids and BP, occurrence of T2DM, Health‐related QoL, Adverse effects (as defined by the authors of the included trials), costs	6mo	Moderate
Shannon, 2020^42^ *Australia, US, UK, Brazil, Sweden, Italy, Spain*	14	Adults aged ≥18y who are healthy or with cardiovascular risk factors (1930)	(I) MedDiet (which was defined as a MedDiet by the authors of each study) administered alone or with other clinical, pharmaceutical, or lifestyle interventions if a comparable and valid control group was included (e.g. MedDiet plus exercise compared with control group including exercise alone) (NR) (C) Swedish diet, Atkins low carbohydrate diet, LF diet, habitual diet, prudent diet, NCEP‐1 diet (NR)	Endothelial function	NR	Low
**Studies of MedDiet in primary and secondary CVD prevention combined**						
Ge, 2020^44^ *US, Australia, Luxembourg, Netherlands, Spain, Italy, France, Greece, Algeria, Germany, Poland, Finland, Iceland, Denmark, Sweden, Brazil, New Zealand, UK, Israel*	30	Adults (≥18y) who were overweight (BMI 25–29 kg/m^2^) or obese (BMI ≥30 kg/m^2^), with MI, T2DM, HTN, sleep apnoea, or cardiovascular risk factors (12 597)	(I) MedDiet (NR) (C) An alternative active or non‐active control diet (e.g. usual) (NR)	Weight loss, blood lipids, BP, or C‐ reactive protein	NR	Critically low
Grosso, 2017^43^ *France, India, Spain, Italy*	4	Adults with high CVD risk and composite cases of CVD (12 293)	(I) MedDiet (7418) (C) Prudent diet, general health advice, LF diet (4875)	CVD events	NR	Critically low
Liyanage, 2016^11^ *France, India, US, UK, HK, Spain*	6	Adults with MI, angina, T2DM, or cardiac risk factors (10 623)	(I) Non‐restricted fat intake and at least two components of Mediterranean style diet as defined by authors (olive oil as main cooking oil, moderate red wine consumption, high consumption of nuts and/or legumes, grains/cereals, fruit and vegetables, fish, and low to moderate consumption of milk and dairy products, low consumption of meat and meat productions) (6605) (C) Any diet (4018)	Total CVD events, Coronary events, Cerebrovascular events, HF, Total mortality, T2DM incidence, End‐stage kidney disease, Adverse effects (all adverse effects and serious adverse effects), QoL	18mo	Moderate
Nissensohn, 2016^45^ *Italy, Spain, Israel, US*	6	Adults with MI, T2DM, obesity, or with cardiovascular risk factors. (7987)	(I) MedDiet (NR) (C) LF diet (NR)	SBP, DBP	NR	Critically low
Rees, 2019^46^ *Australia, US, Brazil, India, Spain, Italy, France, Greece, Poland, China*	30	Adults (≥18y) with and without established CVD (1862)	(I) MedDiet (high intake of plant foods mainly fruits and vegetable, cereals, and whole‐grain breads, beans, nut and seeds, olive oil as main cooking ingredient, low to moderate cheese and yoghurt, low quantities of red meat and higher quantities of fish, low to moderate amounts of red wine) (NR) (C) No intervention, another dietary intervention for primary prevention, usual care, or another dietary intervention for secondary intervention. (NR)	CVD mortality, total mortality, or non‐fatal end points, changes in blood lipids, the occurrence of T2DM, health related QoL, adverse effects, costs	6mo	Moderate

Abbreviations: ADA, American Diabetes Association; BMI, body mass index; BP, blood pressure; CABG, coronary artery bypass graft surgery; CHD, coronary heart disease; C, comparator; DBP, diastolic blood pressure; EU, Europe; FBF, fasting blood glucose; FPG, fasting plasma glucose; GI, glycaemic index; HbA1c, haemoglobin A1c; HDL, high‐density lipoprotein; HK, Hong Kong; HOMA‐IR, homeostatic model assessment of insulin resistance; HTN, hypertension; I, intervention; LC, low carbohydrate; LDL, low‐density lipoprotein; LF, low fat; MetSyn, metabolic syndrome; MI, myocardial infraction; NAFLD, non‐alcoholic fatty liver disease; NCEP‐1, The National Cholesterol Education Program Diet; NR, not reported; Pri, primary; PTCA, percutaneous transluminal coronary angioplasty QoL, quality of life; RCT, randomised control trial; Sec, secondary; SBP, systolic blood pressure; T2DM, type 2 diabetes mellitus; TC, total cholesterol; TG, triglyceride; UK, United Kingdom; US, United States of America; WC, waist circumference; y, years old.

^a^ As described by the systematic review authors; MedDiet has only been used if described as MedDiet, but no further explanation was given in the review.

^b^ Numbers included in intervention and comparator groups within the meta‐analysis.

All 18 reviews with meta‐analyses were evaluated for quality using the AMSTAR‐2 checklist: 11 (61%) reviews were of critically low quality,^31^, ^32^, ^33^, ^34^, ^35^, ^37^, ^38^, ^39^, ^43^, ^44^, ^45^ 4 (22%) reviews were of low quality^13^, ^36^, ^40^, ^42^ and 3 (17%) reviews were of moderate quality.^11^, ^41^, ^46^ No reviews were evaluated as high quality (Table 1 and Supporting Information S1: Table 4). 36% (*n* = 7) reviews did not have a protocol registered prior to the commencement of the review (item 2).^31^, ^34^, ^35^, ^37^, ^43^, ^45^ Only two (11%) reviews had an adequate literature search (item 4),^41^, ^46^ and six (33%) reviews provided a list of excluded studies with explanations of reason (item 7).^11^, ^32^, ^36^, ^40^, ^41^, ^46^ Two (11%) reviews did not include a risk of bias of primary studies included in the review (item 9),^32^, ^43^ and seven (39%) reviews did not consider the risk of bias when interpreting the results (item 13).^31^, ^32^, ^33^, ^37^, ^38^, ^43^ Two (11%) reviews did not have appropriate meta‐analytical methods (item 11).^31^, ^32^ Four (21%) reviews did not assess the presence of likely impact of publication bias (item 15).^31^, ^32^, ^38^, ^45^

Twenty‐eight CVD outcomes for 205 985 participants were categorised into primary (end point) outcomes (Table 2) and surrogate (risk factor) outcomes (Table 3). Primary outcomes were grouped as fatal (*n* = 9) or non‐fatal (*n* = 12). Surrogate outcomes were grouped as vascular measurements (*n* = 20), blood lipids (*n* = 24), blood glucose (*n* = 14) and anthropometric measurements (*n* = 11). The mean age of the participants ranged from 41 to 56 years old. The median age ranged between 51 and 55 years. The age distribution across the reviews showed considerable variability, with ages ranging from 20 to 80 years.

**TABLE 2:** Summary of meta‐analysed results on primary outcomes and CVD risk.

First author, Year (Reference)	N RCTs	Participants Intervention (*n*)^a^	Follow up duration	Effect estimate (95% CI)	Heterogeneity *I* ^2^ (%)	SR quality (AMSTAR‐2)	Certainty of Evidence (GRADE)
*Comparator diet*		Comparator (*n*)					
**Fatal outcomes**							
***All‐cause mortality***							
Rees, 2019^46^ *Another dietary intervention (Pri prevention)*	1	(I) 4997 (C) 2450	4.8y	HR 1.00 (0.80, 1.23)	23.57	Moderate	Low
Rees, 2019^46^ *Another dietary intervention (Sec prevention)*	1	(I) NR (C) NR	2y	RR 0.59 (0.51, 0.68)	NR	Moderate	NR
Rees, 2019^46^ *Usual care (Sec prevention)*	1	(I) 14 (C) 24	46mo	RR 0.44 (0.21, 0.92)	100	Moderate	Low
***CVD‐related mortality***							
Becerra‐Tomas, 2020^13^ *LF, LC*	2	NR	27mo to 4.8y	RR 0.67 (0.45, 1.00)	64	Low	Low
Grosso, 2017^43^ *Prudent, LF diet, general health advice*	4	NR	2–5y	RR 0.59 (0.38, 0.93)	46	Critically low	NR
Liyanage, 2016^11^ ‐	4	(I) 6605 (C) 4018	27mo to 9y	RR 0.90 (0.72, 1.11)	NR	Moderate	NR
Rees, 2019^46^ *Another dietary intervention (Pri prevention)*	1	(I) 2454, 2543 (C) 2450	4.8y	HR 0.81 (0.50, 1.32)	44.57	Moderate	Low
Rees, 2019^46^ *Another dietary intervention (Sec prevention)*	1	(I) NR (C) NR	2y	RR 0.5 (0.42, 0.6)	NR	Moderate	NR
Rees, 2019^46^ *Usual care (Sec prevention)*	1	(I) 6 (C)19	46mo	RR 0.35 (0.15, 0.82)	NR	Moderate	Low
***Cardiac‐related mortality***							
*CHD*							
Becerra‐Tomas, 2020^13^ *LF, LC*	1	NR	2y	RR 0.33 (0.13, 0.85)	NR	Low	Low
*Sudden cardiac death*							
Rees, 2019^46^ *Another dietary intervention (Sec prevention)*	2	(I) NR (C) NR	2y	RR 0.48 (0.37, 0.63)	0	Moderate	NR
*Fatal MI*							
Becerra‐Tomas, 2020^13^ *LF, LC*	1	NR	2y	RR 0.67 (0.31, 1.43)	NR	Low	Very low
Rees, 2019^46^ *Another dietary intervention (Sec prevention)*	2	(I) NR (C) NR	2y	RR 0.66 (0.61, 0.71)	0	Moderate	NR
**Non‐fatal outcomes**							
***CVD‐related events***							
*CVD incidence*							
Becerra‐Tomas, 2020^13^ *LF, LC*	2	NR	27mo‐4.8y	RR 0.62 (0.50, 0.78)	86	Low	Moderate
***Cardiac‐related events***							
*CHD incidence*							
Becerra‐Tomas, 2020^13^ *LF, LC*	1	NR	2y	RR 0.48 (0.33, 0.71)	NR	Low	Low
*Coronary event*							
Liyanage, 2016^11^ ‐	3	(I) 5798 (C) 3254	2–4.8y	RR 0.65 (0.50, 0.85)	NR	Moderate	NR
*Heart failure*							
Liyanage, 2016^11^ ‐	2	(I) 801 (C) 804	2y to 27mo	RR 0.30 (0.17, 0.56)	NR	Moderate	NR
*Non‐fatal MI*							
Becerra‐Tomas, 2020^13^ *LF, LC*	2	NR	2–4.8y	RR 0.65 (0.49, 0.88)	50	Low	Moderate
Grosso, 2017^43^ *Prudent, LF diet, general health advice*	3	NR	2‐5y	RR 0.60 (0.44, 0.82)	26	Critically low	NR
Rees, 2019^46^ *Another dietary intervention (Pri prevention)*	1	(I) 2545, 2546 (C) 2450	4.8y	HR 0.79 (0.57, 1.10)	0	Moderate	Low
Rees, 2019^46^ *Another dietary intervention (Sec prevention)*	1	(I) NR (C) NR	2y	RR 0.47 (0.28, 0.79)	NR	Moderate	NR
***PAD/PVD***							
Rees, 2019^46^ *Another dietary intervention (Pri prevention)*	1	(I) 2454, 2543 (C) 2450	4.8y	HR 0.42 (0.28, 0.61)	5.09	Moderate	Moderate
***Stroke incidence***							
Becerra‐Tomas, 2020^13^ *LF, LC*	1	NR	4.8y	RR 0.58 (0.42, 0.81)	NR	Low	Moderate
Grosso, 2017^43^ *Prudent, LF diet, general health advice*	2	NR	3‐5y	RR 0.64 (0.47, 0.86)	0	Critically low	NR
Liyanage, 2016^11^ ‐	3	(I) 5798 (C) 3254	2–4.8y	RR 0.65 (0.48, 0.88)	NR	Moderate	NR
Rees, 2019^46^ *Another dietary intervention (Pri prevention)*	1	(I) 2454, 2543 (C) 2450	4.8y	HR 0.60 (0.45, 0.80)	0	Moderate	Moderate

Abbreviations: CHD, coronary heart disease; CI, confidence interval; CVD, cardiovascular disease; GRADE, Grading of Recommendations, Assessment, Development, and Evaluations; HR, hazard ratio; LC, low carbohydrate, LF, low fat; MI, myocardial infarction; mo, months; NR, not reported; PAV/PVD, peripheral artery disease/peripheral vascular disease; Pri, primary; RCT, randomised controlled trial; RoB, risk of bias; RR, relative risk; Sec, secondary; SR, systematic review; y, years.

^a^ Numbers included in intervention and comparator groups within the meta‐analysis.

**TABLE 3:** Summary of meta‐analysis results on secondary outcomes and CVD risk.

First author, year (reference) comparator diet	N RCTs	Participants intervention (*n*)^a^ comparator (*n*)	Follow‐up duration	Effect estimate (95% CI)	Heterogeneity *I* ^2^ (%)	RoB of SR (AMSTAR‐2)	Certainty of evidence (GRADE)
***Vascular measurements***							
*Systolic blood pressure reduction (mmHg)*							
Gay, 2016^35^ *Control diet, advice only, or standard follow‐up*	4	NR	NR	MD −1.17 (−2.81, 0.46)	93	Critically low	NR
Huo, 2015^37^ *Usual care, HC, LF, ADA diet*	NR	NR	NR	MD −1.45 (−1.97, −0.94)	0	Critically low	NR
Rees, 2019^46^ *None/minimal (Pri prevention)*	2	(I) 150 (C) 119	3mo to 2y	MD −2.99 (−3.45, −2.53)	0	Moderate	Moderate
Rees, 2019^46^ *Another dietary intervention (Pri prevention)*	4	(I) 229 (C) 219	3mo to 1y	MD −1.50 (−3.92, 0.92)	15.66	Moderate	Low
Rees, 2019^46^ *Usual care (Sec prevention)*	1	(I) 171 (C) 168	4.6y	MD −2 (−5.29, −1.29)	NR	Moderate	Very low
Cowell, 2021^33^ *LF, habitual, prudent, paleo, vegan diet*	19	NR	NR	MD −1.39 (−2.40, −0.39)	53.5	Critically low	NR
Ge, 2020^44^ *Usual diet*	NR	NR	6mo	MdnD 2.95 (0.94, 5.01)	NR	Critically low	Moderate
Ge, 2020^44^ *Usual diet*	NR	NR	12mo	MdnD −2.32 (−5.48,1.13)	NR	Critically low	NR
Ge, 2020^44^ *Dietary advice*	NR	NR	6mo	MdnD 2.37 (5.17, −0.32)	NR	Critically low	Moderate
Ge, 2020^44^ *Dietary advice*	NR	NR	12mo	MdnD −1.04 (−5.03, 3.64)	NR	Critically low	NR
Ge, 2020^44^ *LF*	NR	NR	6mo	MdnD 0.99 (3.58, −1.66)	NR	Critically low	Moderate
Ge, 2020^44^ *LF*	NR	NR	12mo	MdnD −1.33 (2.69, −3.98)	NR	Critically low	NR
Ge, 2020^44^ *Atkins*	NR	NR	6mo	MdnD 2.18 (4.98, −0.65)	NR	Critically low	Moderate
Ge, 2020^44^ *Atkins*	NR	NR	12mo	MdnD 0.35 (4.53, −2.82)	NR	Critically low	NR
Ge, 2020^44^ *DASH*	NR	NR	6mo	MdnD 1.73 (4.27, −0.78)	NR	Critically low	Low
Ge, 2020^44^ *DASH*	NR	NR	12mo	MdnD −6.04 (−1.10, −10.72)	NR	Critically low	NR
Ge, 2020^44^ *Jenny Craig*	NR	NR	6mo	MdnD 4.92 (11.40, −1.62)	NR	Critically low	Low
Ge, 2020^44^ *Jenny Craig*	NR	NR	12mo	MdnD −3.13 (3.72, −9.05)	NR	Critically low	NR
Ge, 2020^44^ *Ornish*	NR	NR	6mo	MdnD −2.26 (1.66, −6.34)	NR	Critically low	Low
Ge, 2020^44^ *Ornish*	NR	NR	12mo	MdnD −3.02 (2.00, −6.97)	NR	Critically low	NR
Ge, 2020^44^ *Palaeolithic*	NR	NR	6mo	MdnD 11.62 (4.22, 19.02)	NR	Critically low	Moderate
Ge, 2020^44^ *Portfolio*	NR	NR	6mo	MdnD 3.02 (−2.46, 8.40)	NR	Critically low	Moderate
Ge, 2020^44^ *Rosemary Conley*	NR	NR	6mo	MdnD −0.56 (4.69, −5.89)	NR	Critically low	Low
Ge, 2020^44^ *The Biggest Loser*	NR	NR	6mo	MdnD 0.23 (−4.88, 5.26)	NR	Critically low	Very low
Ge, 2020^44^ *Volumetrics*	NR	NR	6mo	MdnD −0.02 (−6.64, 6.49)	NR	Critically low	Low
Ge, 2020^44^ *Volumetrics*	NR	NR	12mo	MdnD −1.65 (5.47, −7.96)	NR	Critically low	NR
Ge, 2020^44^ *Weight Watchers*	NR	NR	6mo	MdnD −0.40 (2.57, −3.37)	NR	Critically low	Low
Ge, 2020^44^ *Weight Watchers*	NR	NR	12mo.	MdnD −0.26 (4.56, −4.13)	NR	Critically low	NR
Ge, 2020^44^ *Zone*	NR	NR	6mo	MdnD 0.51 (−2.79, 3.82)	NR	Critically low	Moderate
Ge, 2020^44^ *Zone*	NR	NR	12mo	MdnD −1.34 (−5.19, 3.80)	NR	Critically low	NR
Gibbs, 2021^36^ *Unspecified Referent or Control diet/s*	8	(I) 2698 (C) 2578	NR	MD −0.95 (−1.70, −0.20)	38	Low	Moderate
Ndanuko, 2016^38^ *Usual care, prudent, LF diet*	3	NR	1–2y	MD −3.02 (−3.47, −2.58)	0	Critically low	NR
Nissensohn, 2016^45^ *LF diet*	6	NR	1–2y	MD −1.44 (−2.88, 0.01)	87	Critically low	NR
Papadaki, 2020^40^ *No treatment, usual care, or advice to follow a different diet*	22	NR	NR	MD −1.94 (−2.55, −1.33)	91	Low	NR
*Diastolic blood pressure reduction (mmHg)*							
Gay, 2016^35^ *Control diet, advice only, or standard follow‐up*	4	NR	NR	MD −1.44 (−2.11, −0.76)	82	Critically low	NR
Huo, 2015^37^ *Usual care, HC, LF, ADA diet*	NR	NR	NR	MD −1.41 (−1.84, −0.97)	0	Critically low	NR
Rees, 2019^46^ *None/minimal (Pri prevention)*	2	(I) 150 (C) 119	3mo to 2y	MD −2 (−2.29, −1.71)	0	Moderate	Moderate
Rees, 2019^46^ *Another dietary intervention (Pri prevention)*	4	(I) 229 (C) 219	3mo to 1y	MD −0.26 (−2.41, 1.90)	36.73	Moderate	Low
Rees, 2019^46^ *Usual care (Sec prevention)*	1	(I) 171 (C) 168	4.6y	MD −1 (−4.29, 2.29)	NR	Moderate	Very low
Cowell, 2021^33^ *LF, habitual, prudent, paleo, vegan diet*	19	NR	NR	MD −1.53 (−2.74, −0.32)	71.5	Critically low	NR
Ge, 2020^44^ *Usual Diet*	NR	NR	6mo	MdnD 1.02 (−0.48, 2.53)	NR	Critically low	Moderate
Ge, 2020^44^ *Usual Diet*	NR	NR	12mo	MdnD 1.27 (−1.57, 4.00)	NR	Critically low	NR
Ge, 2020^44^ *Dietary advice*	NR	NR	6mo	MdnD 0.61 (−1.38, 2.64)	NR	Critically low	Moderate
Ge, 2020^44^ *Dietary advice*	NR	NR	12mo	MdnD 2.42 (−1.11, 5.86)	NR	Critically low	NR
Ge, 2020^44^ *LF*	NR	NR	6mo	MdnD −1.22 (0.69, −3.17)	NR	Critically low	Moderate
Ge, 2020^44^ *LF*	NR	NR	12mo	MdnD −0.14 (2.22, −3.32)	NR	Critically low	NR
Ge, 2020^44^ *Atkins*	NR	NR	6mo	MdnD −2.30 (−4.37, −0.25)	NR	Critically low	Moderate
Ge, 2020^44^ *Atkins*	NR	NR	12mo	MdnD −0.51 (−3.74, 2.23)	NR	Critically low	NR
Ge, 2020^44^ *DASH*	NR	NR	6mo	MdnD −1.82 (−3.69, −0.03)	NR	Critically low	Low
Ge, 2020^44^ *DASH*	NR	NR	12mo	MdnD 2.76 (−0.84, 6.23)	NR	Critically low	NR
Ge, 2020^44^ *Jenny Craig*	NR	NR	6mo	MdnD −6.79 (−2.16, −11.45)	NR	Critically low	Low
Ge, 2020^44^ *Jenny Craig*	NR	NR	12mo	MdnD −0.79 (4.11, −6.29)	NR	Critically low	NR
Ge, 2020^44^ *Ornish*	NR	NR	6mo	MdnD 0.82 (3.75, −2.04)	NR	Critically low	Low
Ge, 2020^44^ *Ornish*	NR	NR	12mo	MdnD 1.89 (5.12, −1.82)	NR	Critically low	NR
Ge, 2020^44^ *Palaeolithic*	NR	NR	6mo	MdnD 2.82 (7.87, 2.18)	NR	Critically low	Moderate
Ge, 2020^44^ *Portfolio*	NR	NR	6mo	MdnD −2.96 (−6.83, 0.86)	NR	Critically low	Low
Ge, 2020^44^ *Rosemary Conley*	NR	NR	6mo	MdnD −0.42 (−4.07, 3.24)	NR	Critically low	Very low
Ge, 2020^44^ *The biggest loser*	NR	NR	6mo	MdnD −1.18 (−4.82, 2.47)	NR	Critically low	Low
Ge, 2020^44^ *Volumetrics*	NR	NR	6mo	MdnD −0.92 (3.77, −5.65)	NR	Critically low	Low
Ge, 2020^44^ *Volumetrics*	NR	NR	12mo	MdnD 1.22 (6.44, −4.62)	NR	Critically low	NR
Ge, 2020^44^ *Weight Watchers*	NR	NR	6mo	MdnD −0.02 (2.10, −2.16)	NR	Critically low	Low
Ge, 2020^44^ *Weight Watchers*	NR	NR	12mo	MdnD 1.51 (4.73, −2.15)	NR	Critically low	NR
Ge, 2020^44^ *Zone*	NR	NR	6mo	MdnD −1.31 (−3.77, 1.10)	NR	Critically low	Moderate
Ge, 2020^44^ *Zone*	NR	NR	12mo	MdnD −0.40 (−4.36, 2.73)	NR	Critically low	NR
Gibbs, 2021^36^ *Unspecified referent or control diet*	8	(I) 2698 (C) 2578	NR	MD −0.69 (−1.44, 0.06)	68	Low	Moderate
Ndanuko, 2016^38^ *Usual care, prudent, LF diet*	3	NR	1−2y	MD −1.99 (−2.28, −1.71)	0	Critically low	NR
Nissensohn, 2016^45^ *LF*	6	NR	1–2y	MD −0.70 (−1.34, −0.07)	63	Critically low	NR
Papadaki, 2020^40^ *LF, prudent, usual diet, no diet treatment, ADA, ‘General healthy eating’*	22	NR	NR	MD −0.94 (−1.48, −0.41)	93	Low	NR
*Endothelial function*							
Fatima, 2022^34^ *‐*	19	NR	NR	SMD 0.341 (0.16, 0.52)	72.15	Critically low	NR
Shannon, 2020^42^ *Swedish, Atkins, LF, habitual, prudent, NCEP, SFA diet, non‐MedDiet +exercise*	14	NR	1–27mo	SMD 0.35 (0.17, 0.53)	73.68	Low	NR
*Flow mediated dilation (%)*							
Fatima, 2022^34^ *‐*	10	NR	NR	SMD 1.385 (0.52, 2.25)	88.51	Critically low	NR
Shannon, 2020^42^ *Swedish, Atkins, LF, habitual, prudent, NCEP, SFA diet, non‐MedDiet +exercise*	7	NR	1–27mo	MD 1.66 (1.15, 2.17)	0	Low	NR
Papadaki, 2020^40^ *LF, prudent, usual diet, no diet treatment, ADA, ‘General healthy eating’*	3	NR	NR	MD 1.49 (0.61, 2.37)	0	Low	NR
***Blood lipids (mmol/L)***							
*Total cholesterol*							
Huo, 2015^37^ *Usual care, HC, LF, ADA diet*	6	NR	NR	MD −0.14 (−0.19, −0.09)	0	Critically low	NR
Pan, 2019^39^ *HC*	2	NR	NR	MD −0.01 (−0.40, 0.37)	NR	Critically low	NR
Pan, 2019^39^ LF	4	NR	NR	MD 0.17 (0.08, 0.26)	NR	Critically low	NR
Pan, 2019^39^ *Regular diet*	1	NR	NR	MD 0.01 (−0.69, 0.97)	NR	Critically low	NR
Papadaki, 2020^40^ *LF, prudent, usual diet, no diet treatment, ADA, ‘General healthy eating’*	32	NR	NR	MD −0.23 (−0.34, −0.12)	98	Low	NR
Rees, 2013^41^ *Usual or minimum intervention*	8	(I) 2089 (C) 2062	3mo to 8.1y	MD −0.16 (−0.26, −0.06)	73.58	Moderate	NR
Rees, 2019^46^ *None/minimal (Pri prevention)*	5	(I) 300 (C) 269	1y	MD −0.16 (−0.32, 0)	73.42	Moderate	Low
Rees, 2019^46^ *Another dietary intervention (Pri prevention)*	7	(I) 519 (C) 420	3mo to 4.8y	MD −0.13 (−0.30, 0.04)	70.09	Moderate	Low
Rees, 2019^46^ *Usual care (Sec prevention)*	2	(I) 220 (C) 221	1–4.6y	MD 0.07 (−0.19, 0.33)	19.31	Moderate	Low
Rees, 2019^46^ *Another dietary intervention (Sec prevention)*	2	(I) 655 (C) 628	1–2y	MD −0.5 (−0.61, −0.39)	0	Moderate	NR
*High‐density lipoprotein cholesterol*							
Ajala, 2013^31^ *Other diets*	3	NR	≥6mo	WMD 0.04 (0.01, 0.07)	NR	Critically low	NR
Ge, 2020^44^ *Usual diet*	NR	NR	6mo	MdnD −0.02 (−0.06, 0.02)	NR	Critically low	Low
Ge, 2020^44^ *Usual diet*	NR	NR	12mo	MdnD −0.01 (0.04, −0.07)	NR	Critically low	NR
Ge, 2020^44^ *Dietary advice*	NR	NR	6mo	MdnD 0.03 (−0.03, 0.08)	NR	Critically low	Low
Ge, 2020^44^ *Dietary advice*	NR	NR	12mo	MdnD 0.05 (−0.02, 0.12)	NR	Critically low	NR
Ge, 2020^44^ *LF*	NR	NR	6mo	MdnD 0.04 (−0.01, 0.09)	NR	Critically low	Moderate
Ge, 2020^44^ *LF*	NR	NR	12mo	MdnD 0.11 (0.02, 0.21)	NR	Critically low	NR
Ge, 2020^44^ *Atkins*	NR	NR	6mo	MdnD −0.06 (−0.01, −0.11)	NR	Critically low	Low
Ge, 2020^44^ *Atkins*	NR	NR	12mo	MdnD −0.004 (−0.06, 0.05)	NR	Critically low	NR
Ge, 2020^44^ *DASH*	NR	NR	6mo	MdnD 0.03 (−0.04, 0.1)	NR	Critically low	Low
Ge, 2020^44^ *DASH*	NR	NR	12mo	MdnD 0.1 (0.01, 0.19)	NR	Critically low	NR
Ge, 2020^44^ *Jenny Craig*	NR	NR	6mo	MdnD 0.06 (−0.02, 0.14)	NR	Critically low	Moderate
Ge, 2020^44^ *Jenny Craig*	NR	NR	12mo	MdnD −0.01 (−0.1, 0.08)	NR	Critically low	NR
Ge, 2020^44^ *Ornish*	NR	NR	6mo	MdnD 0.11 (0.19, 0.03)	NR	Critically low	Moderate
Ge, 2020^44^ *Ornish*	NR	NR	12mo	MdnD 0.13 (0.2, 0.05)	NR	Critically low	NR
Ge, 2020^44^ *Palaeolithic*	NR	NR	6mo	MdnD 0.05 (−0.11, 0.21)	NR	Critically low	Low
Ge, 2020^44^ *Portfolio*	NR	NR	6mo	MdnD 0.07 (−0.06, 0.19)	NR	Critically low	Low
Ge, 2020^44^ *Rosemary Conley*	NR	NR	6mo	MdnD 0.04 (−0.09, 0.16)	NR	Critically low	Very low
Ge, 2020^44^ *South beach*	NR	NR	6mo	MdnD −0.03 (−0.19, 0.13)	NR	Critically low	Low
Ge, 2020^44^ *The Biggest Loser*	NR	NR	6mo	MdnD −0.02 (−0.12, 0.09)	NR	Critically low	Very low
Ge, 2020^44^ *Volumetrics*	NR	NR	6mo	MdnD −0.01 (−0.14, 0.12)	NR	Critically low	Low
Ge, 2020^44^ *Volumetrics*	NR	NR	12mo	MdnD 0.03 (0.15, −0.09)	NR	Critically low	NR
Ge, 2020^44^ *Weight Watchers*	NR	NR	6mo	MdnD 0.01 (−0.05, 0.07)	NR	Critically low	Low
Ge, 2020^44^ *Weight Watchers*	NR	NR	12mo	MdnD −0.01 (−0.09, 0.06)	NR	Critically low	NR
Ge, 2020^44^ *Zone*	NR	NR	6mo	MdnD −0.01 (−0.07, 0.06)	NR	Critically low	Low
Ge, 2020^44^ *Zone*	NR	NR	12mo	MdnD 0.06 (−0.01, 0.14)	NR	Critically low	NR
Huo, 2015^37^ *Usual care, HC, LF, ADA diet*	2	NR	NR	MD 0.06 (0.02, 0.10)	53.60	Critically low	NR
Pan, 2019^39^ *HC*	2	NR	NR	MD 0.01 (−0.09, 0.11)	NR	Critically low	NR
Pan, 2019^39^ *LF*	2	NR	NR	MD −0.07 (−0.11, −0.04)	NR	Critically low	NR
Pan, 2019^39^ *Regular diet*	1	NR	NR	MD −0.01 (−0.18, 0.16)	NR	Critically low	NR
Papadaki, 2020^40^ *LF, prudent, usual diet, no diet treatment, ADA, ‘General healthy eating’*	31	NR	NR	MD 0.02 (−0, 0.05)	98	Low	NR
Rees, 2019^46^ *None/minimal (Pri prevention)*	5	(I) 300 (C) 269	3mo–2y	MD 0.02 (−0.04, 0.08)	70.17	Moderate	Low
Rees, 2019^46^ *Another dietary intervention (Pri prevention)*	6	(I) 494 (C) 397	3mo to 4.8y	MD 0.02 (−0.01, 0.04)	0	Moderate	Moderate
Rees, 2019^46^ *Usual care (Sec prevention)*	2	(I) 220 (C) 221	1–4.6y	MD −0.01 (−0.08, 0.07)	12.88	Moderate	Low
Rees, 2019^46^ *Another dietary intervention (Sec prevention)*	3	(I) 692 (C) 662	1–2y	MD 0.06 (−0.01, 0.12)	70.11	Moderate	NR
*Non‐high‐density lipoprotein cholesterol*							
Papadaki, 2020^40^ *LF, prudent, usual diet, no diet treatment, ADA, ‘General healthy eating’*	2	NR	3–6mo	MD 0 (−0.02, 0.01)	60	Low	NR
*Total cholesterol: high‐density lipoprotein ratio*							
Papadaki, 2020^40^ *LF, prudent, usual diet, no diet treatment, ADA, ‘General healthy eating’*	5	NR	1mo–5y	MD −0.03 (−0.08, 0.03)	100	Low	NR
*Low‐density lipoprotein cholesterol*							
Ajala, 2013^31^ *Other diets*	2	NR	≥6mo	WMD −0.08 (−0.24,0.08)	NR	Critically low	NR
Ge, 2020^44^ *Usual diet*	NR	NR	6mo	MdnD 0.11 (0.21, 0.02)	NR	Critically low	Moderate
Ge, 2020^44^ *Usual diet*	NR	NR	12mo	MdnD 0.16 (0.31, 0.01)	NR	Critically low	NR
Ge, 2020^44^ *Dietary advice*	NR	NR	6mo	MdnD 0.17 (0.31, 0.03)	NR	Critically low	Moderate
Ge, 2020^44^ *Dietary advice*	NR	NR	12mo	MdnD 0.24 (0.41, 0.03)	NR	Critically low	NR
Ge, 2020^44^ *LF*	NR	NR	6mo	MdnD 0.07 (0.05, −0.18)	NR	Critically low	Low
Ge, 2020^44^ *LF*	NR	NR	12mo	MdnD 0.18 (0.3, 0.02)	NR	Critically low	NR
Ge, 2020^44^ *Atkins*	NR	NR	6mo	MdnD 0.19 (0.31, 0.06)	NR	Critically low	Low
Ge, 2020^44^ *Atkins*	NR	NR	12mo	MdnD 0.21 (0.35, 0.02)	NR	Critically low	NR
Ge, 2020^44^ *DASH*	NR	NR	6mo	MdnD 0.02 (−0.17, 0.2)	NR	Critically low	Low
Ge, 2020^44^ *DASH*	NR	NR	12mo	MdnD 0.15 (−0.11, 0.4)	NR	Critically low	NR
Ge, 2020^44^ *Jenny Craig*	NR	NR	6mo	MdnD 0.11 (−0.09, 0.31)	NR	Critically low	Low
Ge, 2020^44^ *Jenny Craig*	NR	NR	12mo	MdnD 0.36 (0.57, 0.1)	NR	Critically low	NR
Ge, 2020^44^ *Ornish*	NR	NR	6mo	MdnD −0.01 (−0.2, 0.22)	NR	Critically low	Very low
Ge, 2020^44^ *Ornish*	NR	NR	12mo	MdnD −0.02 (−0.21, 0.21)	NR	Critically low	NR
Ge, 2020^44^ *Palaeolithic*	NR	NR	6mo	MdnD −0.07 (−0.46, 0.31)	NR	Critically low	Very low
Ge, 2020^44^ *Portfolio*	NR	NR	6mo	MdnD −0.44 (−0.15, −0.72)	NR	Critically low	Low
Ge, 2020^44^ *Rosemary Conley*	NR	NR	6mo	MdnD −0.07 (−0.38, 0.24)	NR	Critically low	Very low
Ge, 2020^44^ *South Beach*	NR	NR	6mo	MdnD 0.13 (−0.29, 0.55)	NR	Critically low	Very low
Ge, 2020^44^ *The Biggest Loser*	NR	NR	6mo	MdnD 0.01 (−0.25, 0.28)	NR	Critically low	Very low
Ge, 2020^44^ *Volumetric*	NR	NR	6mo	MdnD −0.07 (−0.25, 0.28)	NR	Critically low	Very low
Ge, 2020^44^ *Volumetric*	NR	NR	12mo	MdnD −0.12 (−0.49, 0.23)	NR	Critically low	NR
Ge, 2020^44^ *Weight Watchers*	NR	NR	6mo	MdnD 0.09 (−0.08, 0.25)	NR	Critically low	Low
Ge, 2020^44^ *Weight Watchers*	NR	NR	12mo	MdnD 0.18 (−0.36, 0.03)	NR	Critically low	NR
Ge, 2020^44^ *Zone*	NR	NR	6mo	MdnD 0.19 (0.03, 0.35)	NR	Critically low	Low
Ge, 2020^44^ *Zone*	NR	NR	12mo	MdnD 0.17 (−0.05, 0.35)	NR	Critically low	NR
Huo, 2015^37^ *Usual care, HC, LF, ADA diet*	5	NR	NR	MD −0.11 (−0.24, 0.01)	0	Critically low	NR
Pan, 2019^39^ *HC*	2	NR	NR	MD 0.07 (−0.24, 0.39)	NR	Critically low	NR
Pan, 2019^39^ *Regular diet*	1	NR	NR	MD 1.11 (0.54, 1.68)	NR	Critically low	NR
Papadaki, 2020^40^ *LF, prudent, usual diet, no diet treatment, ADA, ‘General healthy eating’*	24	NR	NR	MD −0.25 (−0.4, −0.1)	100	Low	NR
Rees, 2013^41^ *Usual or minimum intervention*	6	(I) 1629 (C) 1598	NR	MD −0.07 (−0.13, −0.01)	21.91	Moderate	NR
Rees, 2019^46^ *None/minimal (Pri prevention)*	4	(I) 210 (C) 179	3mo to 2y	MD −0.08 (−0.26, 0.09)	53.72	Moderate	Very low
Rees, 2019^46^ *Another dietary intervention (Pri prevention)*	7	(I) 522 (C) 425	3mo to 4.8y	MD −0.15 (−0.27, −0.02)	46.04	Moderate	Moderate
Rees, 2019^46^ *Usual care (Sec prevention)*	2	(I) 220 (C) 221	1–4.6y	MD 0.11 (−0.09, 0.31)	0	Moderate	Low
*Triglycerides*							
Ajala, 2013^31^ *Other diets*	3	NR	≥6mo	WMD −0.21 (−0.29, −0.14)	NR	Critically low	NR
Huo, 2015^37^ *Usual care, HC, LF, ADA diet*	6	NR	NR	MD −0.29 (−0.47, −0.10)	58.00	Critically low	NR
Pan, 2019^39^ *HC*	2	NR	NR	MD −0.04 (−0.49, 0.42)	NR	Critically low	NR
Pan, 2019^39^ *LF*	2	NR	NR	MD 0.21 (0.16, 0.27)	NR	Critically low	NR
Pan, 2019^39^ *Regular diet*	1	NR	NR	MD 0.04 (−0.61, 0.69)	NR	Critically low	NR
Papadaki, 2020^40^ *LF, prudent, usual diet, no diet treatment, ADA, ‘General healthy eating’*	33	NR	NR	MD −0.15 (−0.19, −0.11)	94	Low	NR
Rees, 2019^46^ *Another dietary intervention (Pri prevention)*	7	(I) 519 (C) 420	3mo to 4.8y	MD −0.09 (−0.16, −0.01)	14.68	Moderate	Moderate
Rees, 2019^46^ *Usual care (Sec prevention)*	2	(I) 220 (C) 221	1–4.6y	MD −0.14 (−0.38, 0.1)	0	Moderate	Low
***Blood glucose***							
*HbA1c (%)*							
Ajala, 2013^31^ *Usual Care, ADA*	3	(I) 308 (C) 280	NR	WMD −0.41 (−0.58, −0.24)	82	Critically low	NR
Carter, 2014^32^ *Paleo diet*	NR	NR	NR	WMD 0.22 (−0.05, 0.49)	NR	Critically low	NR
Carter, 2014^32^ *LF*	NR	NR	NR	WMD 0.30 (−0.01, 0.61)	NR	Critically low	NR
Carter, 2014^32^ *Healthy diet*	NR	NR	NR	WMD −0.31 (−0.61, −0.01)	NR	Critically low	NR
Huo, 2015^37^ *Usual care, HC, LF, ADA diet*	9	NR	NR	MD −0.30 (−0.46, −0.14)	67.2	Critically low	NR
Pan, 2019^39^ *HC*	4	NR	NR	MD 0.10 (−0.31, 0.51)	NR	Critically low	NR
Pan, 2019^39^ *LF*	1	NR	NR	MD 0.45 (0.34, 0.55)	NR	Critically low	NR
Pan, 2019^39^ *Regular diet*	1	NR	NR	MD −0.28 (−0.54, −0.02)	NR	Critically low	NR
Papadaki, 2020^40^ *LF, prudent, usual diet, no diet treatment, ADA, ‘General healthy eating’*	5	NR	NR	MD −0.29 (−0.40, −0.18)	4	Low	NR
*Fasting BG/PG (mmol/L)*							
Carter, 2014^32^ *Paleo diet*	NR	NR	NR	WMD 1.10 (0.22, 1.98)	NR	Critically low	NR
Carter, 2014^32^ *LF*	NR	NR	NR	WMD 0.03 (−0.12,0.19)	NR	Critically low	NR
Carter, 2014^32^ *Healthy diet*	NR	NR	NR	WMD 0.10 (−0.08, 0.28)	NR	Critically low	NR
Huo, 2015^37^ *Usual care, HC, LF, ADA diet*	6	NR	NR	MD −0.72 (−1.24, −0.21)	66.1	Critically low	NR
Pan, 2019^39^ *HC*	2	NR	NR	MD 0.32 (−0.58, 1.21)	NR	Critically low	NR
Pan, 2019^39^ *LF*	2	NR	NR	MD 1.24 (0.91, 1.57)	NR	Critically low	NR
Pan, 2019^39^ *Regular diet*	1	NR	NR	MD 0.10 (−1.74, 1.94)	NR	Critically low	NR
Papadaki, 2020^40^ *LF, prudent, usual diet, no diet treatment, ADA, ‘General healthy eating’*	28	NR	NR	MD −0.17 (−0.26, −0.07)	98	Low	NR
*Fasting Insulin (μU/mL)*							
Carter, 2014^32^ *Paleo diet*	NR	NR	NR	WMD 2.16 (−2.56, 6.88)	NR	Critically low	NR
Carter, 2014^32^ *LF*	NR	NR	NR	WMD 2.63 (2.05, 3.21)	NR	Critically low	NR
Carter, 2014^32^ *Healthy diet*	NR	NR	NR	WMD −0.60 (−2.18, 0.98)	NR	Critically low	NR
Huo, 2015^37^ *Usual care, HC, LF, ADA diet*	5	NR	NR	MD −0.55 (−0.81, −0.29)	0	Critically low	NR
Papadaki, 2020^40^ *LF, prudent, usual diet, no diet treatment, ADA, ‘General healthy eating’*	20	NR	NR	MD −0.94 (−1.72, −0.16)	97	Low	NR
*HOMA‐IR*							
Papadaki, 2020^40^ *LF, prudent, usual diet, no diet treatment, ADA, ‘General healthy eating’*	16	NR	NR	MD −0.41 (−0.70, −0.12)	98	Low	NR
*T2DM incidence*							
Rees, 2019^46^ *Another dietary intervention (Pri prevention)*	1	(I) 2394 (C) 1147	4.8y	HR 0.71 (0.52, 0.96)	47.02	Moderate	NR
***Anthropometric measurements***							
*Weight loss (kg)*							
Ajala, 2013^31^ *Control diet*	3	NR	NR	WMD −1.84 (−2.45, −1.15)	NR	Critically low	NR
Ge, 2020^44^ *Usual diet*	NR	NR	6mo	MdnD 2.87 (4.2, 1.6)	NR	Critically low	Moderate
Ge, 2020^44^ *Usual diet*	NR	NR	12mo	MdnD 2.80 (4.72, 0.86)	NR	Critically low	NR
Ge, 2020^44^ *Dietary advice*	NR	NR	6mo	MdnD 2.57 (4.23, 0.94)	NR	Critically low	Moderate
Ge, 2020^44^ *Dietary advice*	NR	NR	12mo	MdnD 3.43 (5.32, 1.56)	NR	Critically low	NR
Ge, 2020^44^ *LF*	NR	NR	6mo	MdnD −0.90 (−2.31, 0.56)	NR	Critically low	Moderate
Ge, 2020^44^ *LF*	NR	NR	12mo	MdnD 0.14 (1.57, −1.37)	NR	Critically low	NR
Ge, 2020^44^ *Atkins*	NR	NR	6mo	MdnD −2.59 (−4.16, −0.98)	NR	Critically low	Low
Ge, 2020^44^ *Atkins*	NR	NR	12mo	MdnD −1.04 (−2.77, 0.65)	NR	Critically low	NR
Ge, 2020^44^ *DASH*	NR	NR	6mo	MdnD −0.76 (−2.34, 0.87)	NR	Critically low	Very low
Ge, 2020^44^ *DASH*	NR	NR	12mo	MdnD −0.27 (−3.51, 2.94)	NR	Critically low	NR
Ge, 2020^44^ *Jenny Craig*	NR	NR	6mo	MdnD −4.90 (−7.33, −2.39)	NR	Critically low	Low
Ge, 2020^44^ *Jenny Craig*	NR	NR	12mo	MdnD −4.38 (−6.84, −1.91)	NR	Critically low	NR
Ge, 2020^44^ *Ornish*	NR	NR	6mo	MdnD 0.77 (−1.76, 3.30)	NR	Critically low	Moderate
Ge, 2020^44^ *Ornish*	NR	NR	12mo	MdnD −0.45 (2.03, −2.79)	NR	Critically low	NR
Ge, 2020^44^ *Palaeolithic*	NR	NR	6mo	MdnD 2.44 (−0.85, 5.77)	NR	Critically low	Low
Ge, 2020^44^ *Palaeolithic*	NR	NR	12mo	MdnD 4.18 (0.33, 8.07)	NR	Critically low	NR
Ge, 2020^44^ *Portfolio*	NR	NR	6mo	MdnD 0.77 (−3.68, 5.16)	NR	Critically low	Very low
Ge, 2020^44^ *Rosemary Conley*	NR	NR	6mo	MdnD 0.89 (3.78, −1.99)	NR	Critically low	NR
Ge, 2020^44^ *Rosemary Conley*	NR	NR	12mo	MdnD −0.01 (2.76, − 2.69)	NR	Critically low	Very low
Ge, 2020^44^ *Slimming world*	NR	NR	6mo	MdnD −0.72 (2.42, −3.90)	NR	Critically low	Low
Ge, 2020^44^ *Slimming world*	NR	NR	12mo	MdnD −1.53 (1.66, −4.65)	NR	Critically low	NR
Ge, 2020^44^ *South beach*	NR	NR	6mo	MdnD 6.99 (2.66, 11.28)	NR	Critically low	Moderate
Ge, 2020^44^ *The biggest loser*	NR	NR	6mo	MdnD 0.01 (−3.59, 3.52)	NR	Critically low	Very low
Ge, 2020^44^ *Volumetrics*	NR	NR	6mo	MdnD 3.08 (−1.04, 7.18)	NR	Critically low	Low
Ge, 2020^44^ *Volumetrics*	NR	NR	12mo	MdnD 1.36 (−2.43, 5.19)	NR	Critically low	NR
Ge, 2020^44^ *Weight watchers*	NR	NR	6mo	MdnD 1.03 (−0.76, 2.77)	NR	Critically low	Low
Ge, 2020^44^ *Weight watchers*	NR	NR	12mo	MdnD 0.18 (−1.76, 2.10)	NR	Critically low	NR
Ge, 2020^44^ *Zone*	NR	NR	6mo	MdnD 1.20 (−1.59, −2.97)	NR	Critically low	Low
Ge, 2020^44^ *Zone*	NR	NR	12mo	MdnD 0.45 (−1.52, 2.49)	NR	Critically low	NR
*Body weight (kg)*							
Huo, 2015^37^ *Usual care, HC, LF, ADA diet*	6	NR	1mo to 4y	MD −0.29 (−0.55, −0.04)	0	Critically low	NR
Papadaki, 2020^40^ *LF, prudent, usual diet, no diet treatment, ADA, ‘General healthy eating’*	36	NR	NR	MD −1.69 (−2.40, −0.98)	99	Low	NR
Pan, 2019^39^ *LF*	4	NR	NR	MD 1.18 (0.37, 1.99)	NR	Critically low	NR
Pan, 2019^39^ *HC*	2	NR	NR	MD 0.24 (−5.49, 5.97)	NR	Critically low	NR
*BMI (kg/m* ^*2*^ *)*							
Huo, 2015^37^ *Usual care, HC, LF, ADA diet*	6	NR	1mo to 4y	MD −0.29 (−0.46, −0.12)	0	Critically low	NR
Pan, 2019^39^ *HC*	1	NR	NR	MD 0.10 (−2.15, 2.35)	NR	Critically low	NR
Pan, 2019^39^ *LF*	2	NR	NR	MD −6.30 (−1.29, 0.02)	NR	Critically low	NR
Pan, 2019^39^ *Regular diet*	1	NR	NR	MD 0.30 (−1.87, 2.47)	NR	Critically low	NR
Papadaki, 2020^40^ *LF, prudent, usual diet, no diet treatment, ADA, ‘General healthy eating’*	32	NR	10d to 5y	MD −0.43 (−0.76, −0.10)	99	Low	NR
*WC (cm)*							
Pan, 2019^39^ *HC*	1	NR	NR	MD 0.00 (−4.48, 4.48)	NR	Critically low	NR
Pan, 2019^39^ *LF*	4	NR	NR	MD 0.73 (0.19, 1.26)	NR	Critically low	NR
Papadaki, 2020^40^ *LF, prudent, usual diet, no diet treatment, ADA, ‘General healthy eating’*	24	NR	1.5mo to 4.8y	MD −0.94 (−2.08, 0.19)	100	Low	NR
*Total Fat Mass (kg)*							
Papadaki, 2020^40^ *LF, prudent, usual diet, no diet treatment, ADA, ‘General healthy eating’*	9	NR	1–12mo	MD −0.47 (−1.53, 0.60)	85	Low	NR
*Total Body Fat (%)*							
Papadaki, 2020^40^ *LF, prudent, usual diet, no diet treatment, ADA, ‘General healthy eating’*	8	NR	2–12mo	MD −0.12 (−1.60, 1.37)	90	Low	NR

Abbreviations: ADA, American Diabetes Association diet; BMI, body mass index; CI, confidence interval; cm, centimetres; DASH, Dietary Approaches to Stop Hypertension; Fasting BG/PG, fasting blood glucose/plasma glucose; GRADE, Grading of Recommendations, Assessment, Development, and Evaluations; HbA1c, haemoglobin A1c; HC, high carbohydrates; HR, hazard ratio; HOMA‐IR, Homeostatic Model Assessment for Insulin Resistance; kg, kilograms; kg/m^2^, kilogram per square metre; LF, low fat; MD, mean difference; MdnD, median difference; mmHg, millimetres of mercury; mmol/L, millimoles per litre; mo, months; NCEP, National Cholesterol Education Program; NR, not reported; Pri, primary; RCT, randomised controlled trial; RoB, risk of bias; Sec, secondary; SFA, saturated fatty acid; SMD, standard mean difference; SR, systematic review; T2DM, type 2 diabetes mellitus; μU/mL, micro‐International Unit per millilitre; WC, waist circumference; WMD, weighted mean difference; y, years.

^a^ Numbers included in intervention and comparator groups within the meta‐analysis.

One systematic review meta‐analysed two studies for the effectiveness of the MedDiet on all‐cause mortality.^46^ The review found low‐certainty evidence that MedDiet reduced all‐cause mortality rates by approximately 50% among patients with a history of CVD (i.e. secondary prevention) with relative risk estimates of 0.44 (95% CI: 0.21–0.92) when compared to usual care. One RCT in the same review reported a relative risk of 0.59 (95% CI: 0.51–0.68) when compared with another active dietary intervention but was excluded from the main analysis due to published expressions of concern.^47^ However, the MedDiet was not associated with statistically significant reductions in all‐cause mortality among adults without a history of CVD (i.e. primary prevention).^46^

**FIGURE 2:**
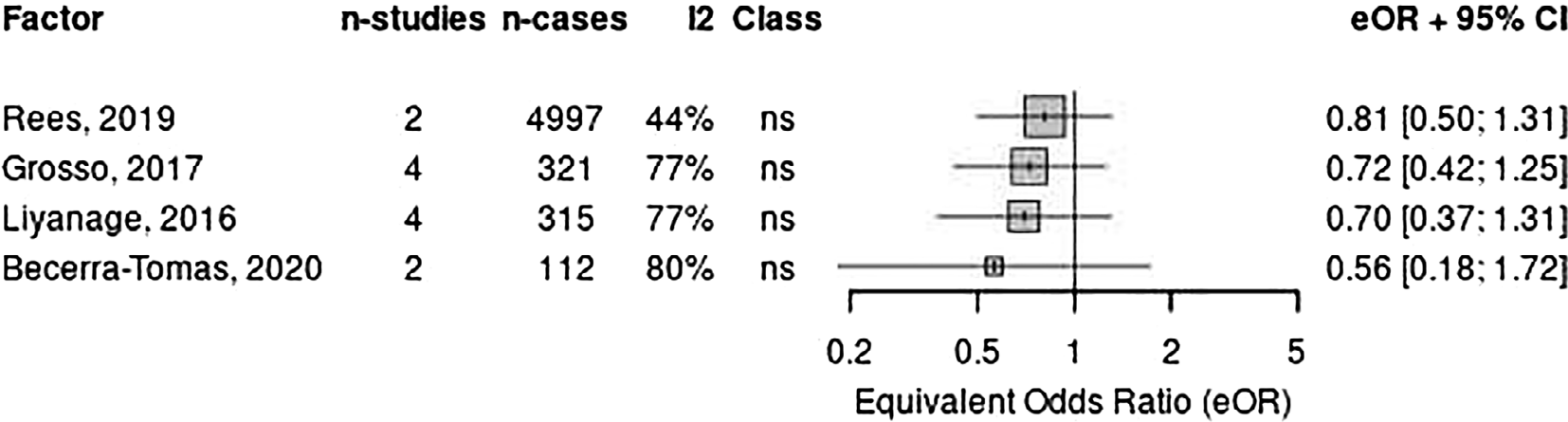
Meta‐analysis of two or more systematic reviews (data from individual RCTs reported) for CVD deaths. CVD, cardiovascular disease; RCT, randomised control trial.

Four reviews^11^, ^13^, ^43^, ^46^ of the 13 RCTs provided limited evidence that the MedDiet reduced CVD‐related mortality (Figure 2) by a range of 10% (four RCTs; RR: 0.9; 95% CI: 0.72–1.11; moderate quality)^11^ to 65% (one RCT; RR: 0.35; 95% CI: 0.15–0.82; moderate quality).^46^ One of these reviews was of critically low quality.^43^ Similarly, two of these reviews^13^, ^46^ of six RCTs also reported on cardiac‐related mortality (i.e. death from CHD, sudden cardiac death and fatal myocardial infarction); however, only one of these reviews included more than one MedDiet RCT. The larger review found that fatal myocardial infarction was reduced by 34% (two RCTs; RR: 0.66; 95% CI: 0.61–0.71; moderate quality)^46^ when comparing MedDiet with another dietary intervention, with the other review (one RCT; RR: 0.67; 95% CI: 0.31–1.43; low quality) showing no significance when the MedDiet was compared with a low‐fat or low‐carbohydrate control group.^13^ Risk of sudden cardiac death was reduced by 52% when the MedDiet was compared to another dietary intervention (two RCTs; RR: 0.48; 95% CI: 0.37–0.63; moderate quality).^46^ One review with one RCT showed a 67% reduced risk of death from CHD (RR: 0.33; 95% CI: 0.13–0.85; low quality)^13^; however, the RCT reported on is one with a published expression of concern.^48^, ^49^ Overall, the findings reported on cardiac‐related mortality were inconsistent and associated with a low to very low certainty of evidence (Table 2).

**FIGURE 3:**
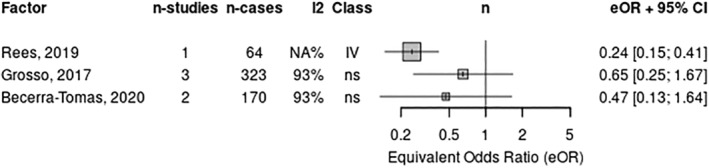
Meta‐analysis of two or more systematic reviews (data from individual RCTs reported) for non‐fatal MI. MI, myocardial infraction; RCT, randomised control trial.

Regarding non‐fatal outcomes, one review included two RCTs and found that the MedDiet reduced CVD incidence by 38% (RR: 0.62; 95% CI: 0.50–0.78; low quality) compared to a low‐fat diet.^13^ Four systematic reviews with a total of 13 RCTs showed that the MedDiet reduced cardiac‐related events (i.e. CHD incidence, coronary events, heart failure and non‐fatal MI).^11^, ^13^, ^43^, ^46^ One of these was evaluated as a critically low‐quality review.^43^ For coronary events and non‐fatal myocardial infarction (Figure 3), the reduction in non‐fatal events associated with the MedDiet ranged from none/non‐significant (one RCT; HR: 0.79; 95% CI: 0.57–1.10, moderate quality) to 53% (one RCT; RR: 0.47; 95% CI: 0.28–0.79; moderate quality) when compared with another dietary intervention.^46^ For heart failure, the reduction was 70% (two RCTs; RR: 0.30; 95% CI: 0.17–0.56; moderate quality) when compared with any diet.^11^ Only one review reported on peripheral artery disease (peripheral vascular disease; Table 2).^46^

**FIGURE 4:**
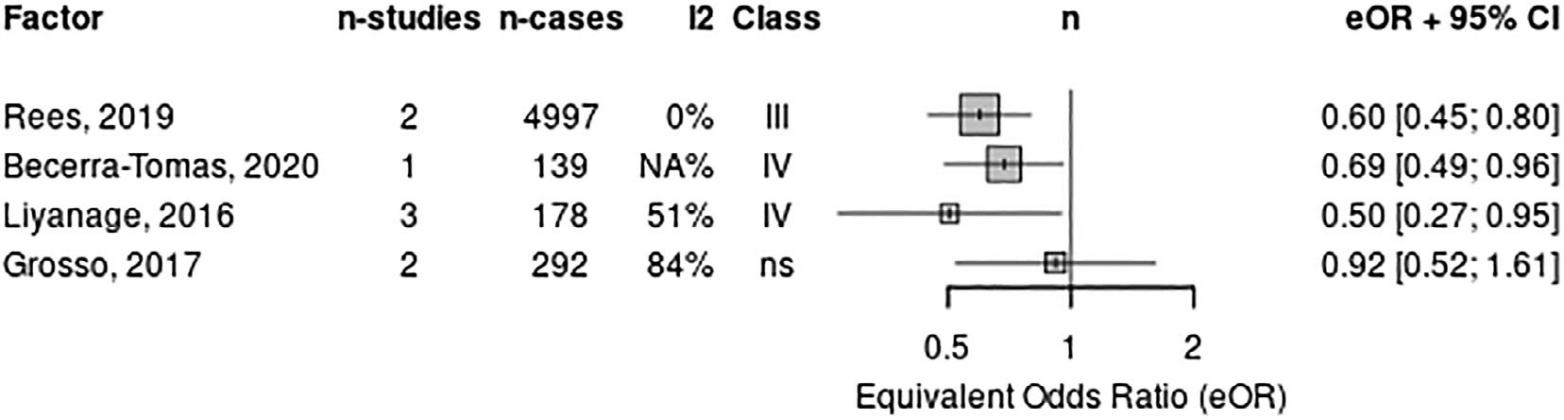
Meta‐analysis of two or more systematic reviews (data from individual RCTs reported) for stroke. RCT, randomised control trial.

Stroke incidence (Figure 4) was reported on in four reviews^11^, ^13^, ^43^, ^46^ comprising seven RCTs. One of these was a critically low‐quality review.^43^ The MedDiet reduced stroke incidence consistently across all four reviews by a range of 35% (three RCTs; RR: 0.65; 95% CI: 0.48–0.88; moderate quality)^11^ to 42% (one RCT; RR: 0.58; 95% CI: 0.42–0.81, low quality).^13^ Overall, the MedDiet was shown to have a beneficial effect on both fatal and non‐fatal CVD outcomes. The GRADE certainty of the evidence was moderate (Table 2).

Seven of the nine systematic reviews that meta‐analysed the effect of the MedDiet on systolic blood pressure (SBP; Table 3) found that the MedDiet reduced SBP by 1–5 mmHg.^33^, ^36^, ^37^, ^38^, ^40^, ^44^, ^46^ Only three of these reviews were not of critically low quality, with one being assessed as moderate quality,^46^ and the other two assessed as low quality.^36^, ^40^ Results of one review which meta‐analysed two RCTs found that the MedDiet was significantly more effective at lowering SBP than no/minimal intervention (−2.99 mmHg; 95% CI: −3.45 to −2.53) for the primary prevention of CVD (moderate‐certainty evidence).^46^ Of the two low‐quality reviews, one compared the MedDiet to unspecified referent/control diets in eight included‐RCTs,^36^ finding that SBP in the MedDiet groups had lower values (MD: −0.95; 95% CI: −1.70 to −0.20); the other compared the MedDiet to a variety of comparator diets or no intervention and reported stronger findings of benefit (MD: −1.94; 95% CI: −2.55 to −1.33).^40^ Three critically low‐quality meta‐analyses comprising 41 RCTs also reported that the MedDiet significantly reduced SBP levels by between 1.39 mmHg (95% CI: −2.40 to −0.39) and 3.02 mmHg (95% CI: −3.47 to −2.58) compared to other diets,^33^, ^37^, ^38^ or usual care,^37^, ^38^ but none undertook GRADE assessments so did not report the certainty of evidence. In contrast, one review (number of RCTs not reported) that evaluated the MedDiet of 6–12 months duration against various other diets found that the Palaeolithic diet improved SBP significantly more than MedDiet at 6 months (median difference: 11.6 mmHg; 95% CI: 4.22–19.02).^44^ This review was assessed as critically low quality, but the GRADE extracted from the review indicated moderate certainty regarding the effect estimate (Table 3). Seven of the nine included reviews reporting blood pressure showed that the MedDiet reduced diastolic blood pressure (DBP), with a mean difference ranging from −0.70 (95% CI: −1.34 to −0.07) to −2.00 (95% CI: −2.29 to −1.71) mmHg.^33^, ^35^, ^37^, ^38^, ^40^, ^45^, ^46^ Of these seven reviews, only one was assessed as being of moderate quality,^46^ and it reported lower DBP in the MedDiet group compared with no or minimal control diet across two RCTs focused on primary prevention (MD: −2.00 mmHg; 95% CI: −2.29 to −1.71; moderate certainty). This same review reported on one RCT, which compared MedDiet with usual care for secondary prevention with a mean lower DBP in the MedDiet group (MD: −1.00; 95% CI: −4.29 to −2.29; very low certainty).^46^ The remaining six reviews were assessed as either low quality^40^ or critically low quality and all six did not assess GRADE (Table 3).^33^, ^35^, ^37^, ^38^, ^45^

Five of the seven reviews reporting HbA1c found that the MedDiet was associated with a greater reduction in HbA1c compared to control diets in adults with type 2 diabetes and high risk for CVD (Table 3).^31^, ^32^, ^37^, ^39^, ^40^ None of these reviews assessed certainty of evidence, and all but one of these reviews were assessed as critically low quality. The exception was a review of five RCTs comparing the MedDiet to various comparator diets, usual diet, and no diet treatment, and was assessed as low quality. The meta‐analysis showed a lower % HbA1C for MedDiet groups (MD: −0.29%; 95% CI: −0.40 to −0.18) compared to various comparator diets, usual diet and no diet treatment.^40^

Four (60%)^40^, ^41^, ^44^, ^46^ of the seven reviews reported that the MedDiet reduced low‐density lipoprotein cholesterol (LDL) levels. One review inclusive of 24 RCTs showed a significant reduction of 0.25 mmol/L (95% CI: −0.4 to −0.1) LDL favouring the MedDiet compared to various other diets but with considerable heterogeneity.^40^ This was a low‐quality review with uncertainty surrounding the effect estimate. Another review also showed low certainty evidence of a significant median difference in LDL levels (−0.44; 95% CI: −0.15 to −0.72) after a 6‐month MedDiet intervention compared to the Portfolio diet.^44^ This review was of critically low quality and did not provide sufficient information regarding the number of RCTs assessed and their characteristics. Two reviews by a different research group revealed a significant LDL reduction with the MedDiet.^41^, ^46^ The first review comprising six RCTs, showed a 0.07 mmol/L (95% CI: −0.13 to −0.01) reduction in LDL in favour of the MedDiet compared to usual or minimal intervention diets.^41^ The second review consisting of seven RCTs, showed a significant reduction in LDL of 0.15 mmol/L (95% CI: −0.27 to −0.02) favouring the MedDiet against other diets for the primary prevention of CVD.^46^ Both meta‐analyses were of moderate quality; however, showed uncertainty around effect estimates due to very low certainty or no GRADE reporting (Table 3).

Three^31^, ^37^, ^44^ of the six reviews that meta‐analysed the effects of the MedDiet on high‐density lipoprotein cholesterol (HDL) showed that the MedDiet was effective in increasing HDL levels in adults with type 2 diabetes and at high risk of CVD. Two reviews comprising five RCTs showed a significant increase in HDL ranging from 0.06 mmol/L (95% CI: 0.02−0.10) to 0.04 mmol/L (95% CI: 0.01−0.07) compared to various diets.^31^, ^37^ Both studies were of critically low quality, with no GRADE reporting, and one review reported substantial heterogeneity, thus introducing uncertainty surrounding the effect estimates. Another review that meta‐analysed the effects of HDL at 12 months showed a significant difference in median HDL of 0.10 mmol/L (95% CI: 0.01–0.19) compared to the DASH diet and 0.11 mmol/L (95% CI: 0.02–0.21) compared to a low‐fat diet.^44^ MedDiet was also favoured compared to the Ornish diet in this review at 6 and 12 months.^44^ The critically low‐quality review did not provide details regarding the number of RCTs analysed, nor was a GRADE assessment conducted, thus reducing confidence regarding the certainty of effect estimates (Table 3).

Three reviews meta‐analysed the effects of the MedDiet and change in BMI compared to various other diets.^37^, ^39^, ^40^ Only one low‐quality review that meta‐analysed 32 RCTs found a statistically significant reduction (MD: 0.43 kg/m^2^, 95% CI: −0.76 to −0.10).^40^ The review reported considerably high levels of heterogeneity, and a need for GRADE, thus lowering the certainty of evidence regarding the effect estimates (Table 3).

## 4 DISCUSSION

This umbrella review aimed to review the previously synthesised evidence on the effect of the MedDiet on all‐cause mortality and 28 CVD‐related outcomes, including primary outcomes such as fatal and non‐fatal outcomes and surrogate outcomes such as vascular measurements, blood lipids, blood glucose, and anthropometry. An additional 10 meta‐analysed reviews met the inclusion criteria in the current umbrella review, published since the earlier 2017 umbrella review that had included CVD outcomes among other health outcomes and which did not focus exclusively on RCTs.^19^ The overall analysis comprised 18 meta‐analyses of RCTs, with a total of 197 965 participants. Most summary estimates identified evidence that supported the beneficial effects of the MedDiet for those at high risk of CVD (particularly individuals with type 2 diabetes), or in the secondary prevention of CVD (i.e. in those with a history of CVD), for primary outcomes including CVD mortality, myocardial infarction, and particularly stroke, most strongly in individuals with type 2 diabetes. The systematic reviews included in our review had relatively few SLRs of interventions investigating the impact of MedDiet on secondary prevention. There were five systematic reviews (16 secondary prevention RCTs) captured within our results. Early results from a recently published large secondary prevention RCT (CORDIOPREV), demonstrating the positive impacts of MedDiet for secondary prevention of CVD, were captured in two of the systematic reviews included in our umbrella review despite its recent publication date.^34^, ^42^ However, the most recent findings from this study, with a full sample of *n* = 1000, were not included.^16^ The large sample size, robust trial design and positive findings suggest that future meta‐analyses that include this trial will result in more certainty around the evidence for MedDiet and secondary prevention of CVD. For surrogate outcomes, the evidence suggests that the MedDiet may improve blood pressure, blood glucose, and lipids. The quality and certainty of evidence for both primary and secondary prevention of CVD ranged from critically low to moderate. The certainty of the evidence fluctuated between limited (no GRADE) and moderate.

Although all the SLRs included in this review reported data from RCTs conducted in both Mediterranean and non‐Mediterranean countries, there remains a potential gap in the evidence about the effectiveness and acceptability of MedDiet interventions in non‐Mediterranean countries. However, studies in non‐Mediterranean countries are growing and it is possible that the level of support (counselling, resources and individualisation) may improve acceptability in non‐Mediterranean regions. Importantly, intervention studies conducted in Mediterranean countries will have baseline dietary intakes much more aligned with the MedDiet to begin with. Effective adherence to control diets in these countries is problematic, and highly cited RCTs have been criticised for the similarity between MedDiet intervention and control group dietary intakes, particularly regarding macronutrients, including fatty acid profiles.^18^, ^50^ The findings of our umbrella review are consistent with previous studies showing an inverse relationship between the MedDiet and CVD risk, whereby the MedDiet reduced CVD risk.^19^, ^51^, ^52^ A high intake of nuts, fruits, vegetables and legumes, with moderate fish intake and high polyphenol olive oil as the main dietary fat, have been proposed to be key characteristics of the MedDiet that confer these benefits.^51^, ^52^ Although individual components of the MedDiet are beneficial,^48^ our review highlights the overarching benefits of the MedDiet as a dietary pattern. This pattern is reflective of all components and highlights the synergistic effects of this dietary pattern, including all food groups and varieties, which provides a more nuanced understanding of the pattern of eating.^49^ Additionally, we speculate that lifestyle or pattern of eating approaches to diet, such as a prescribed MedDiet intervention, may be easier to adhere to long term than proscriptive or restrictive diets. Several studies demonstrate that greater adherence to the MedDiet reduces the risk of primary outcomes such as CVD mortality, CVD incidence, CHD, stroke risk and surrogate outcomes such as blood lipids, blood glucose and anthropometric measures.^19^, ^53^, ^54^ This reinforces the potential benefit of using a lifestyle‐based dietary pattern, like the MedDiet, to manage primary and secondary CVD risk.

Nutrition guidelines are often criticised for the inclusion of observational evidence without well‐conducted RCTs, often resulting in almost every outcome being linked to one or more dietary components.^21^ The inclusion of reviews reporting RCTs in the current review provides support for dietary guidance for CVD prevention in Australia. For example, the findings support the 2019 Heart Foundation of Australia's recommendations, which promote healthy dietary patterns, including the main components of the MedDiet, for lowering CVD risk.^55^, ^56^ These guidelines emphasise plant‐based foods and replacing saturated and trans fats with dietary monounsaturated and polyunsaturated fats to improve CVD outcomes but do not specify extra virgin olive oil as the primary nutritional fat component. The Australian Dietary Guidelines are currently under review for the first time since 2013,^57^ and this may represent an opportunity to further move away from nutrient‐based recommendations to true food‐based recommendations.

This umbrella review has several strengths and limitations. A strength of the current review is the comprehensiveness of the search. We searched five databases, including the dissertations database ProQuest, to identify potentially unpublished reviews in PhD theses with neutral or negative findings, in an effort to minimise publication bias. We also considered the overlap in the primary studies across the included reviews, which identifies any overrepresentation of results where trials are included in more than one review. Additionally, if reported by included reviews, GRADE assessments were extracted and recorded. Providing certainty of evidence ratings can aid in clinical decision‐making. However, 44% of outcomes did not have GRADE applied, and we did not conduct new GRADE assessments, thus reducing the certainty of evidence where GRADE was not conducted within the original review. We did not report on intervention compliance or adherence in our review. The included meta‐analyses did not consistently report on characteristics regarding included MedDiet interventions and most did not report on adherence to the dietary interventions within their included RCTs, so this was not able to be extracted and comprehensively assessed. This is a limitation of our review but also reflects the available published reviews that met our inclusion criteria. Our decision to only include reviews reporting RCTs as a preferred study design to establish causality, we acknowledge that the nature of RCTs for dietary interventions, known to be complex, may limit the generalisability of our findings. Another limitation is that we only included reviews that conducted a meta‐analysis and excluded potentially relevant and better‐quality reviews that only reported narratively summarised results. However, arguably the absence of meta‐analysis in potentially includable systematic reviews is an indicator of less robust review methodology itself.

While our review supports the use of the MedDiet as a dietary pattern intervention for CVD prevention through RCT evidence, our quality appraisals of the included reviews suggest issues with the quality of published reviews and substantial heterogeneity within some of the meta‐analyses reported. The primary studies of the included systematic reviews compared the MedDiet with a wide range of diets, such as DASH, low fat and prudent diet, or with no dietary intervention or usual care. Although we aimed to provide an up‐to‐date umbrella review of the effects of MedDiet compared to any other diet, the variability of included comparators contributes to heterogeneity. These variations should be taken into consideration when evaluating the results of the study. In addition, several important outcomes, such as CHD mortality and CHD incidence, were only analysed by one review making the results inconclusive for these outcomes. As noted, the included systematic reviews failed to report adherence to the dietary protocols of the RCTs reported.

Future reviews should consist of a meta‐analysis of outcomes where multiple studies report the same outcome, as the lack of meta‐analysis resulted in the exclusion of many reviews. Editors and journal reviewers of systematic reviews could provide feedback to authors where meta‐analyses are possible but not conducted.

This umbrella review employed meta‐regression analysis and narratively summarised the results of 18 meta‐analyses on the effectiveness of MedDiet on CVD prevention. Most of the included reviews were of critically low quality. This review showed that the MedDiet can reduce fatal CVD outcome risk by 10%–67% and non‐fatal CVD outcome risk by 21%–70%. This preventive effect was more significant in studies that included populations with a history of CVD (secondary prevention) than those in only people at risk of CVD (primary prevention).

## AUTHOR CONTRIBUTIONS

LA conceived the study; LH, FM, YL, DR, HO, HM and LA contributed to the protocol development and search strategy; LH, FM and YL screened search results; LH, FM and YL extracted data and conducted quality appraisals of included studies; DR, HO, HM, HG and LA resolved conflicts with study selection, data extraction and quality appraisal; LH, FM, and YL led data synthesis and drafting of the manuscript; DR, HO, HM, HG and LA revised the manuscript; DR HO and LA were responsible for supervision and approval of the final content; all authors read and approved the final manuscript. The authors acknowledge Justin Clark (Research Enhancement Manager) and Sarah Bateup (Health Sciences and Medicine Faculty Librarian) for their assistance and expertise in refining the search strategy.

## CONFLICT OF INTEREST STATEMENT

Dianne Reidlinger is Editor of Nutrition & Dietetics. She was excluded from the peer review process and all decision‐making regarding this article. The Journal's Editor‐in‐Chief has managed this manuscript throughout the review process. The Journal operates a blinded peer review process, and the peer reviewers for this manuscript were unaware of the authors of the manuscript. This process prevents authors who also hold an editorial role to influence the editorial decisions made. The authors report no other conflicts of interest.

## Supporting information


**Data S1.** Supporting Information.

## Data Availability

Data sharing not applicable ‐ no new data generated.
