# Supplementary material for: The effectiveness of the Mediterranean Diet for primary and secondary prevention of cardiovascular disease: An umbrella review
Source: Nutr Diet. 2024 Aug 14;82(1):8–41. doi: 10.1111/1747-0080.12891 (PMC11795232; doi:10.1111/1747-0080.12891)
Supplement: Supplementary file 1 — Data S1. Supporting Information. [file NDI-82-8-s001.docx]

**Supplementary Material**

Supplementary Table 1. Electronic Database Search Strategies

Searches executed on 04 November 2022; total number of records retrieved: 2651

| **Pubmed**  904 results    ("Cardiovascular Diseases"[Mesh] OR "Cardiovascular disease"[tiab] OR "CVD"[tiab] OR "Heart disease"[tiab] OR Heart[tiab] OR Mortality[tiab] OR "All-cause mortality"[tiab] OR "Cardiovascular mortality"[tiab] OR "Coronary heart disease"[tiab] OR "CHD"[tiab]OR Coronary[tiab] OR "Myocardial infarction"[tiab] OR "MI"[tiab] OR Stroke[Mesh] OR Stroke[tiab] OR Strokes[tiab] OR "Ischemic heart disease"[tiab] OR "Haemorrhagic stroke"[tiab] OR Haemorrhagic[tiab] OR Haemorrhage[tiab] OR "Ischemic stroke"[tiab] OR Myocardial[tiab] OR Myocarditis[tiab] OR Myocardium[tiab] OR Cholesterol[Mesh] OR Lipoprotein[tiab] OR "Blood pressure"[Mesh] OR "Blood pressure"[tiab] OR Hypertension[tiab] OR Hypertensive[tiab] OR "Diabetes Mellitus"[Mesh] OR Diabetes[tiab] OR Diabetic[tiab] OR "Metabolic syndrome"[tiab] OR "Insulin resistance"[Mesh])  AND  ("Diet, Mediterranean"[Mesh] OR "Mediterranean diet"[tiab] OR "Mediterranean style diet"[tiab] OR "Mediterranean dietary pattern"[tiab] OR "MedDiet"[tiab] OR "Dietary pattern"[tiab] OR "Mediterranean food"[tiab] OR "Olive oil"[tiab] OR Fish[tiab] OR ((Vegetable[tiab] OR Vegetables[tiab]) AND (Legume[tiab] OR Legumes[tiab]) AND (Nuts[tiab]) AND (Grain[tiab] OR Grains[tiab])))  AND  (Prevention[tiab] OR Prevent[tiab] OR Prevents[tiab] OR Risk[tiab] OR Risks[tiab])  AND  ("Meta analysis"[pt] OR "Systematic review"[pt] OR ((Search[tiab] OR Searched[tiab]) AND (PubMed[tiab] OR MEDLINE[tiab])) OR (Systematic[tiab] AND Review[tiab]) OR "Meta analysis"[tiab] OR Meta-analysis[tiab] OR Review[ti] OR ((Systematically[tiab] OR Reviewed[tiab]) AND (literature[tiab])) OR "Cochrane Database Syst Rev"[jour]) |
| --- |
| **The Cochrane Database of Systematic Reviews**  28 Results  ([mh "Cardiovascular Diseases"] OR "Cardiovascular disease":ti,ab OR CVD:ti,ab OR "Heart disease":ti,ab OR Heart:ti,ab OR Mortality:ti,ab OR "All-cause mortality":ti,ab OR "Cardiovascular mortality":ti,ab OR "Coronary heart disease":ti,ab OR CHD:ti,ab OR Coronary:ti,ab OR "Myocardial infarction":ti,ab OR MI:ti,ab OR [mh Stroke] OR Stroke:ti,ab OR Strokes:ti,ab OR "Ischemic heart disease":ti,ab OR "Haemorrhagic stroke":ti,ab OR Haemorrhagic:ti,ab OR Haemorrhage:ti,ab OR "Ischemic stroke":ti,ab OR Myocardial:ti,ab OR Myocarditis:ti,ab OR Myocardium:ti,ab OR [mh Cholesterol] OR Lipoprotein:ti,ab OR [mh "Blood pressure"] OR "Blood pressure":ti,ab OR Hypertension:ti,ab OR Hypertensive:ti,ab OR [mh "Diabetes Mellitus"] OR Diabetes:ti,ab OR Diabetic:ti,ab OR "Metabolic syndrome":ti,ab OR [mh "Insulin resistance"])  AND  ([mh "Diet, Mediterranean"] OR "Mediterranean diet":ti,ab OR "Mediterranean style diet":ti,ab OR "Mediterranean dietary pattern":ti,ab OR MedDiet:ti,ab OR "Dietary pattern":ti,ab OR "Mediterranean food":ti,ab OR "Olive oil":ti,ab OR Fish:ti,ab OR ((Vegetable:ti,ab OR Vegetables:ti,ab) AND (Legume:ti,ab OR Legumes:ti,ab) AND (Nuts:ti,ab) AND (Grain:ti,ab OR Grains:ti,ab)))  AND  (Prevention:ti,ab OR Prevent:ti,ab OR Prevents:ti,ab OR Risk:ti,ab OR Risks:ti,ab) |
| **Embase**  1355 results    ('Cardiovascular Diseases'/exp OR 'Cardiovascular disease':ti,ab OR CVD:ti,ab OR 'Heart disease':ti,ab OR Heart:ti,ab OR Mortality:ti,ab OR 'All-cause mortality':ti,ab OR 'Cardiovascular mortality':ti,ab OR 'Coronary heart disease':ti,ab OR CHD:ti,ab OR Coronary:ti,ab OR 'Myocardial infarction':ti,ab OR MI:ti,ab OR Stroke/exp OR Stroke:ti,ab OR Strokes:ti,ab OR 'Ischemic heart disease':ti,ab OR 'Haemorrhagic stroke':ti,ab OR Haemorrhagic:ti,ab OR Haemorrhage:ti,ab OR 'Ischemic stroke':ti,ab OR Myocardial:ti,ab OR Myocarditis:ti,ab OR Myocardium:ti,ab OR Cholesterol/exp OR Lipoprotein:ti,ab OR 'Blood pressure'/exp OR 'Blood pressure':ti,ab OR Hypertension:ti,ab OR Hypertensive:ti,ab OR 'Diabetes Mellitus'/exp OR Diabetes:ti,ab OR Diabetic:ti,ab OR 'Metabolic syndrome':ti,ab OR 'Insulin resistance'/exp)  AND  ('Diet, Mediterranean'/exp OR 'Mediterranean diet':ti,ab OR 'Mediterranean style diet':ti,ab OR 'Mediterranean dietary pattern':ti,ab OR MedDiet:ti,ab OR 'Dietary pattern':ti,ab OR 'Mediterranean food':ti,ab OR 'Olive oil':ti,ab OR Fish:ti,ab OR ((Vegetable:ti,ab OR Vegetables:ti,ab) AND (Legume:ti,ab OR Legumes:ti,ab) AND (Nuts:ti,ab) AND (Grain:ti,ab OR Grains:ti,ab)))  AND  (Prevention:ti,ab OR Prevent:ti,ab OR Prevents:ti,ab OR Risk:ti,ab OR Risks:ti,ab)  AND  ([cochrane review]/lim OR [systematic review]/lim OR [meta analysis]/lim OR ((Search:ti,ab OR Searched:ti,ab) AND (PubMed:ti,ab OR MEDLINE:ti,ab)) OR (Systematic:ti,ab AND Review:ti,ab) OR 'Meta analysis':ti,ab OR Meta-analysis:ti,ab OR Review:ti OR ((Systematically:ti,ab OR Reviewed:ti,ab) AND (literature:ti,ab))) |
| **CINAHL**  333 results    ((MH "Cardiovascular Diseases+") OR (TI "Cardiovascular disease" OR AB "Cardiovascular disease") OR (TI CVD OR AB CVD) OR (TI "Heart disease" OR AB "Heart disease") OR (TI Heart OR AB Heart) OR (TI Mortality OR AB Mortality) OR (TI "All-cause mortality" OR AB "All-cause mortality") OR (TI "Cardiovascular mortality" OR AB "Cardiovascular mortality") OR (TI "Coronary heart disease" OR AB "Coronary heart disease") OR (TI CHD OR AB CHD) OR (TI Coronary OR AB Coronary) OR (TI "Myocardial infarction" OR AB "Myocardial infarction") OR (TI MI OR AB MI) OR (MH Stroke+) OR (TI Stroke OR AB Stroke) OR (TI Strokes OR AB Strokes) OR (TI "Ischemic heart disease" OR AB "Ischemic heart disease") OR (TI "Haemorrhagic stroke" OR AB "Haemorrhagic stroke") OR (TI Haemorrhagic OR AB Haemorrhagic) OR (TI Haemorrhage OR AB Haemorrhage) OR (TI "Ischemic stroke" OR AB "Ischemic stroke") OR (TI Myocardial OR AB Myocardial) OR (TI Myocarditis OR AB Myocarditis) OR (TI Myocardium OR AB Myocardium) OR (MH Cholesterol+) OR (TI Lipoprotein OR AB Lipoprotein) OR (MH "Blood pressure+") OR (TI "Blood pressure" OR AB "Blood pressure") OR (TI Hypertension OR AB Hypertension) OR (TI Hypertensive OR AB Hypertensive) OR (MH "Diabetes Mellitus+") OR (TI Diabetes OR AB Diabetes) OR (TI Diabetic OR AB Diabetic) OR (TI "Metabolic syndrome" OR AB "Metabolic syndrome") OR (MH "Insulin resistance+"))  AND  ((MH "Diet, Mediterranean+") OR (TI "Mediterranean diet" OR AB "Mediterranean diet") OR (TI "Mediterranean style diet" OR AB "Mediterranean style diet") OR (TI "Mediterranean dietary pattern" OR AB "Mediterranean dietary pattern") OR (TI MedDiet OR AB MedDiet) OR (TI "Dietary pattern" OR AB "Dietary pattern") OR (TI "Mediterranean food" OR AB "Mediterranean food") OR (TI "Olive oil" OR AB "Olive oil") OR (TI Fish OR AB Fish) OR (((TI Vegetable OR AB Vegetable) OR (TI Vegetables OR AB Vegetables)) AND ((TI Legume OR AB Legume) OR (TI Legumes OR AB Legumes)) AND ((TI Nuts OR AB Nuts)) AND ((TI Grain OR AB Grain) OR (TI Grains OR AB Grains))))  AND  ((TI Prevention OR AB Prevention) OR (TI Prevent OR AB Prevent) OR (TI Prevents OR AB Prevents) OR (TI Risk OR AB Risk) OR (TI Risks OR AB Risks))  AND  (((MH "Meta analysis")) OR ((TI "Meta analys*" OR AB "Meta analys*")) OR ((TI Metaanaly* OR AB Metaanaly*)) OR ((MH "Literature review+")) OR (((TI systematic OR AB systematic) W1 ((TI review OR AB review) OR (TI overview OR AB overview))))) NOT ((PT Commentary) OR (PT Letter) OR (PT Editorial) OR ((MH "Animals"))) |
| **Pro-Quest**  31results  (MESH.EXACT.EXPLODE("Cardiovascular Diseases") OR TI,AB("Cardiovascular disease") OR TI,AB(CVD) OR TI,AB("Heart disease") OR TI,AB(Heart) OR TI,AB(Mortality) OR TI,AB("All-cause mortality") OR TI,AB("Cardiovascular mortality") OR TI,AB("Coronary heart disease") OR TI,AB(CHD) OR TI,AB(Coronary) OR TI,AB("Myocardial infarction") OR TI,AB(MI) OR MESH.EXACT.EXPLODE(Stroke) OR TI,AB(Stroke) OR TI,AB(Strokes) OR TI,AB("Ischemic heart disease") OR TI,AB("Haemorrhagic stroke") OR TI,AB(Haemorrhagic) OR TI,AB(Haemorrhage) OR TI,AB("Ischemic stroke") OR TI,AB(Myocardial) OR TI,AB(Myocarditis) OR TI,AB(Myocardium) OR MESH.EXACT.EXPLODE(Cholesterol) OR TI,AB(Lipoprotein) OR MESH.EXACT.EXPLODE("Blood pressure") OR TI,AB("Blood pressure") OR TI,AB(Hypertension) OR TI,AB(Hypertensive) OR MESH.EXACT.EXPLODE("Diabetes Mellitus") OR TI,AB(Diabetes) OR TI,AB(Diabetic) OR TI,AB("Metabolic syndrome") OR MESH.EXACT.EXPLODE("Insulin resistance"))  AND  (MESH.EXACT.EXPLODE("Diet, Mediterranean") OR TI,AB("Mediterranean diet") OR TI,AB("Mediterranean style diet") OR TI,AB("Mediterranean dietary pattern") OR TI,AB(MedDiet) OR TI,AB("Dietary pattern") OR TI,AB("Mediterranean food") OR TI,AB("Olive oil") OR TI,AB(Fish) OR ((TI,AB(Vegetable) OR TI,AB(Vegetables)) AND (TI,AB(Legume) OR TI,AB(Legumes)) AND (TI,AB(Nuts)) AND (TI,AB(Grain) OR TI,AB(Grains))))  AND  (TI,AB(Prevention) OR TI,AB(Prevent) OR TI,AB(Prevents) OR TI,AB(Risk) OR TI,AB(Risks))  AND  (NOFT("Meta analysis") OR NOFT("Systematic review") OR ((TI,AB(Search) OR TI,AB(Searched)) AND (TI,AB(PubMed) OR TI,AB(MEDLINE))) OR (TI,AB(Systematic) AND TI,AB(Review)) OR TI,AB("Meta analysis") OR TI,AB(Meta-analysis) OR TI(Review) OR ((TI,AB(Systematically) OR TI,AB(Reviewed)) AND (TI,AB(literature))) OR "Cochrane Database Syst Rev[jour]" ) |

Supplementary Table 2: List of excluded studies with reasons

| **Reason for Exclusion** | **References** |
| --- | --- |
| **Did not include a meta-analysis (n=42)** | **Only systematic reviews** (Ajala 2018; Abbate 2020; Adegbola 2022; Bayes 2022; Bloomfield 2016; Cole 2011; English 2021; Esposito 2010; Esposito 2015; Francis 2019; García-Fernández 2014; García-Fernández 2019; García Pérezde de Sevilla 2022; Grao-Cruces 2021; Grosso 2014; Iacoviello 2018; Kastorini 2010; Klonizakis 2021; Luong 2022; Maderuelo-Fernandez 2015; Maghsoudi 2012; Mancini 2016; Martinez-Gonzalez 2014; Maruthur 2014; Padwal 2016; Papamichou 2019; Roman 2008; Sanches Machado d’Almeida 2018; Sánchez-Sánchez 2020; Serra-Majem 2006; Silveira 2021; SimõesCorrêaGalendi 2022; Sleiman 2015; Sotos-Prieto 2010) |
| **Wrong study design (n=56)**  Reason not reported (n=38)  Umbrella review (n =3)  Not published (n =1)  Systematic review of narrative reviews (n=2)  Abstract only (n= 11)  Review of Abstract (n=1) | **Wrong study design** (Garcia 2016; Salas-Salvadó 2016; Pérez-López 2009; Tyrovolas 2010;Chen 2019; Hosseini 2016; Medina-Remón 2018; Riccardi 2022;Vilela 2021; Hu 2002; Tuncay 2020; Psaltopoulou 2013; Khazrai 2014; Zyriax 2022; Eleftheriou 2018; Chiva-Blanch 2014; VanHorn 2008; Hooper 2007; Foroughi 2013; AlAufi 2022; Kahleova 2019; DosReisPadilha 2018; Khemayanto 2014; Sofi 2008; Chareonrungrueangchai 2020; DePergola 2018; Toi 2020; Gomez-Delgado 2022; Ahluwalia 2013; Mead 2006; Kargin 2019; Chu 2016; Ros 2014; GarcíaPastor 2021)  **Umbrella review** (Dinu 2018; Dinu 2020; Khan 2021)  **Not published** (Pezzuoli 2021)  **Narrative review:** (Saulle 2019; Wermers 2020)  **Abstract only:** (Bach-Faig 2018; Lara 2015; Hernandez 2020; Doundoulakis 2021; Panagiotakos 2014; Stranges 2019; Park 2015; Nordmann 2011; PafferFilho 2019; Kerley 2018; Francis 2017)  **Review of abstract** (Havranek 2011) |
| **Wrong intervention** **(n = 9)**  Not reported (n =4)  Dash (n=1)  Low fat diet (n=1)  MedDiet + exercise (n =1)  Methodology of MedDiet (n=1) | **Not reported:** (D'Alessandro 2019; Uusitupa 2019; Defagó 2013; Hartley 2013)  **DASH:** (Aljefree 2015)  **Low fat diet:** (Sherzai 2012)  **MedDiet + exercise:** (Malakou 2018)  **Methodology of MedDiet:** (Huedo-Medina 2016) |
| **Wrong outcomes (n=4)** | (Neale 2016; Deichert 2018, Mayr 2018, Kastorini 2011) |
| **Same study different author (n=1)** | (Boushey 2020 – Same as English 2021) |

Supplementary Table 3: Meta-analysed studies excluded for reporting only one RCT

| Review Excluded | RCTs Included | Comment |
| --- | --- | --- |
| Kontogianni 2014 | Estruch et al., 2013 (Included in this umbrella review already) | Also included MA of Psaltopoulou et al, 2012 which did not include any RCTs |
| Schwingshackl 2015 | Salas-Salvado et al., 2014 (Included in this umbrella review already) |  |
| Wan, 2022 | Estruch et al., 2018 (Included in this umbrella review already) |  |
| Koloverou 2014 | Salas-Salvado et al., 2011 ((Included in this umbrella review already) |  |
| Jannasch 2017 | Salas-Salvado et al, 2014 (Included in this umbrella review already) |  |
| Francis 2019 * MA |  | The SLR included was part of a PhD thesis and it was not possible to determine eligibility. |

Supplementary Table 4. Quality appraisal results of included meta-analyses using the AMSTAR-2 Tool.

| First Author, Year | Q1 | Q2 | Q3 | Q4 | Q5 | Q6 | Q7 | Q8 | Q9 | Q10 | Q11 | Q11 | Q13 | Q14 | Q15 | Q16 | Overall rating |
| --- | --- | --- | --- | --- | --- | --- | --- | --- | --- | --- | --- | --- | --- | --- | --- | --- | --- |
| Ajala, 2013 | Y | N | N | N | N | N | N | PY | Y | N | N | N | N | Y | N | Y | Critically Low |
| Becerra-Tomas, 2020 | Y | Y | Y | PY | Y | Y | N | PY | Y | Y | Y | Y | Y | Y | Y | Y | Low |
| Carter, 2014 | Y | PY | Y | PY | Y | Y | Y | PY | N | N | N | N | N | Y | N | Y | Critically Low |
| Cowell, 2021 | Y | Y | Y | PY | Y | Y | N | PY | Y | N | Y | N | N | Y | Y | Y | Critically low |
| Fatima, 2020 | Y | N | Y | N | Y | Y | N | PY | Y | N | Y | Y | Y | Y | Y | Y | Critically Low |
| Gay, 2016 | Y | N | Y | PY | Y | N | N | PY | Y | N | Y | Y | N | N | Y | Y | Critically Low |
| Ge, 2020 | Y | Y | y | N | Y | Y | N | PY | Y | Y | Y | Y | Y | Y | Y | Y | Critically Low |
| Gibbs, 2021 | Y | Y | N | N | N | Y | Y | PY | Y | Y | Y | Y | Y | Y | Y | Y | Low |
| Grosso, 2017 | N | N | N | N | Y | N | N | PY | N | N | Y | N | N | Y | Y | N | Critically Low |
| Huo et al., 2015 | Y | N | N | N | Y | N | N | PY | Y | N | Y | N | N | Y | Y | Y | Critically Low |
|  |  |  |  |  |  |  |  |  |  |  |  |  |  |  |  |  |  |
| Liyanage, 2016 | Y | PY | Y | PY | Y | Y | Y | PY | Y | N | Y | Y | Y | N | Y | Y | Moderate |
| Ndanuko, 2016 | Y | Y | Y | N | N | N | N | PY | Y | N | Y | N | N | Y | N | Y | Critically Low |
| Nissensohn, 2016 | Y | N | Y | PY | Y | N | N | Y | Y | Y | Y | Y | Y | Y | N | Y | Critically Low |
| Pan, 2019 | Y | Y | Y | N | y | y | N | PY | Y | N | Y | Y | Y | Y | Y | Y | Critically Low |
| Papadaki, 2020 | Y | Y | N | N | Y | Y | Y | PY | Y | N | Y | Y | Y | Y | Y | Y | Low |
| Rees, 2019 | Y | Y | Y | Y | Y | Y | Y | Y | Y | N | Y | N | Y | Y | Y | Y | Moderate |
| Rees, 2013 | Y | Y | Y | Y | Y | Y | Y | Y | Y | N | Y | N | Y | Y | Y | Y | Moderate |
| Shannon, 2020 | Y | Y | Y | PY | Y | Y | N | PY | Y | N | Y | Y | Y | Y | Y | Y | Low |

Y: yes; N: no; PY: partial yes

Q1: **Did the research questions and inclusion criteria for the review include the components of PICO?**

Q2: **Did the report of the review contain an explicit statement that the review methods were established prior to the conduct of the review and did the report justify any significant deviations from the protocol?**

Q3: **Did the review authors explain their selection of the study designs for inclusion in the review?**

Q4: **Did the review authors use a comprehensive literature search strategy?**

Q5: **Did the review authors perform study selection in duplicate?**

**Q6:** **Did the review authors perform data extraction in duplicate?**

**Q7:** **Did the review authors provide a list of excluded studies and justify the exclusions?**

**Q8:** **Did the review authors describe the included studies in adequate detail?**

**Q9:** **Did the review authors use a satisfactory technique for assessing the risk of bias (RoB) in individual studies that were included in the review?**

**Q10: Did the review authors report on the sources of funding for the studies included in the review?**

**Q11: If meta-analysis was performed did the review authors use appropriate methods for statistical combination of results?**

**Q12:** **If meta-analysis was performed, did the review authors assess the potential impact of RoB in individual studies on the results of the meta-analysis or other evidence synthesis?**

**Q13:** **Did the review authors account for RoB in individual studies when interpreting/ discussing the results of the review?**

**Q14:** **Did the review authors provide a satisfactory explanation for, and discussion of, any heterogeneity observed in the results of the review?**

**Q15: If they performed quantitative synthesis did the review authors carry out an adequate investigation of publication bias (small study bias) and discuss its likely impact on the results of the review?**

**Q16: Did the review authors report any potential sources of conflict of interest, including any funding they received for conducting the review**
